# Multi‐omic analysis in carcinoma of unknown primary (CUP): therapeutic impact of knowing the unknown

**DOI:** 10.1002/1878-0261.13293

**Published:** 2023-11-16

**Authors:** Shumei Kato, Sophia Gumas, Jacob J. Adashek, Ryosuke Okamura, Suzanna Lee, Jason K. Sicklick, Razelle Kurzrock

**Affiliations:** ^1^ Center for Personalized Cancer Therapy and Division of Hematology and Oncology, Department of Medicine UC San Diego Moores Cancer Center La Jolla CA USA; ^2^ Department of Oncology, The Sidney Kimmel Comprehensive Cancer Center The Johns Hopkins Hospital Baltimore MD USA; ^3^ Department of Surgery Kyoto University Hospital Japan; ^4^ Division of Surgical Oncology, Department of Surgery UC San Diego School of Medicine CA USA

**Keywords:** carcinoma of unknown primary, next‐generation sequencing, precision oncology, targeted therapy

## Abstract

Carcinoma of unknown primary (CUP) is a difficult‐to‐manage malignancy. Multi‐omic profiles and treatment outcome vs. degree of precision matching were assessed. Tumours underwent next‐generation sequencing (NGS) [tissue and/or blood‐derived cell‐free DNA (cfDNA)]. Selected patients had transcriptome‐based immune profiling and/or programmed cell death 1 ligand 1 (PD‐L1) immunohistochemistry analysis. Patients could be reviewed by a Molecular Tumor Board, but physicians chose the therapy. Of 6497 patients in the precision database, 97 had CUP. The median number of pathogenic tissue genomic alterations was 4 (range, 0–25), and for cfDNA, was 2 (range, 0–9). Each patient had a distinct molecular landscape. Food and Drug Administration (FDA)‐approved biomarkers included the following: PD‐L1^+^ ≥ 1%, 30.9% of CUPs tested; microsatellite instability, 3.6%; tumour mutational burden ≥ 10 mutations·Mb^−1^, 23%; and neurotrophic receptor tyrosine kinase (NTRK) fusions, 0%. RNA‐based immunograms showed theoretically druggable targets: lymphocyte activation gene 3 protein (LAG‐3), macrophage colony‐stimulating factor 1 receptor (CSF1R), adenosine receptor A2 (ADORA2) and indoleamine 2,3‐dioxygenase 1 (IDO1). Overall, 56% of patients had ≥ 1 actionable biomarker (OncoKB database). To quantify the degree of matching (tumours to drugs), a Matching Score (MS; roughly equivalent to number of alterations targeted/total number of deleterious alterations) was calculated *post hoc*. Comparing evaluable treated patients [MS high, > 50% (*N* = 15) vs. low ≤ 50% (*N* = 47)], median progression‐free survival was 10.4 vs. 2.8 months (95% CI 0.11–0.64; HR 0.27; *P* = 0.002); survival, 15.8 vs. 6.9 months (95% CI 0.17–1.16; HR 0.45; *P* = 0.09); and clinical benefit rate (stable disease ≥ 6 months/partial/complete response), 71% vs. 24% (*P* = 0.003). Higher MS was the only factor that predicted improvement in outcome variables after multivariate analysis. In conclusion, CUPs are molecularly complex. Treatments with high degrees of matching to molecular alterations (generally achieved by individualized combinations) correlated with improved outcomes.

## Graphical abstract

To improve outcomes in patients with carcinomas of unknown primary, a multiomic approach is needed including genomic, transcriptomic, immunomic and proteomic insights.

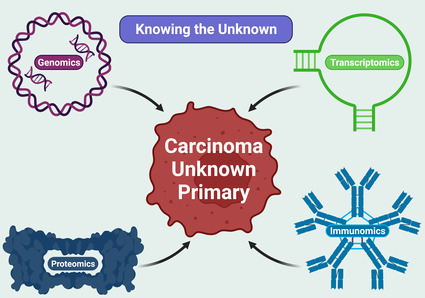

**Abbreviations**
cfDNA: cell‐free DNA
CI: confidence interval
CLIA: clinical laboratory improvement amendments
CR: complete response
CUP: carcinoma of unknown primary
FDA: food and drug administration
HR: Hazard ratio
IC_50_
: Inhibitory concentration 50%
MS: matching score
MSI‐H: microsatellite instability‐high
NGS: next‐generation sequencing
OR: odds ratio
OS: overall survival
PD: progressive disease
PD‐L1: programmed death‐ligand 1
PFS: progression‐free survival
PR: partial response
PREDICT: profile related evidence determining individualized cancer therapy
ROC: receiver operating characteristic curve
RR: response rate
SD: stable disease
TMB: tumour mutational burden
UCSD: University of California, San Diego
VUS: variants of unknown significance

## 1 Introduction

With an incidence of 7–12 cases per 100 000 per year [1], carcinoma of unknown primary (CUP) is an uncommon cancer. Carcinoma of unknown primary is a diagnosis of exclusion wherein no primary can be identified in a metastatic setting, despite a comprehensive workup with serum biomarker analysis, imaging and histologic evaluations with various immunohistochemistry analyses [2, 3, 4]. Because of the lack of a diagnosis, treating CUP is exceedingly difficult and there is not substantial evidence regarding the best approach. Historically, patients have received platinum‐based combination chemotherapy regimens, with reported response rates (RRs) of 20–40% and median overall survival (OS) of 6–8 months [5, 6].

A few studies have evaluated the utility of gene expression profiling‐based site‐specific therapy vs. empiric chemotherapy. However, the phase III GEFCAPI 04 trial, which randomized patients with CUP (*N* = 243) to either cisplatin/gemcitabine or tailored therapy based on predicted primary site of cancer by gene expression profiling, failed to improve median progression‐free survival (PFS; 5.3 vs. 4.6 months; *P* = 0.7) and OS [10 vs. 10.7 months; hazard ratio (HR) 0.92, 0.69–1.23] [7]. Hayashi et al. [8] also randomized 130 CUP patients to empiric paclitaxel/carboplatin or gene expression‐based site‐specific therapy; however, there was no survival difference. To improve the dismal outcome, investigation with immune checkpoint inhibitors is underway, with preliminary results suggesting moderate clinical efficacy with pembrolizumab [anti‐programmed cell death‐1 (PD‐1) inhibitor] among CUP patients [partial response (PR) rate of 23% (3/13 patients)] [9].

To better understand the molecular characteristics of CUP and to identify potentially actionable alterations, next‐generation sequencing (NGS) of both tissue and cell‐free DNA (cfDNA) has been evaluated [10, 11, 12, 13]. Patients with CUP have alterations in multiple genes, including *TP53* (38–55% of patients), *KRAS* (18–20%), *CDKN2A* (19%), *MYC* (12%), *ARID1A* (11%) and *PIK3CA* (9–14%) [10, 11, 12, 13]. In order to evaluate whether or not targeting genomic alterations can improve the clinical outcome, basket/umbrella trials are ongoing in patients with CUP (e.g. the CUPISCO trial evaluating standard treatment vs. genomically targeted therapy based on molecular profiling) [14]. However, a limitation of many precision oncology trials is that their strategy is often based on targeting one gene at a time, in the presence of a tumour sample having multiple aberrations. This issue is especially important in devising a treatment approach for patients with CUP, since most of these malignancies have a median of about four pathogenic genomic abnormalities per tumour [10, 12, 13].

We hypothesized that agents or combinations that impacted more vs. less actionable alterations would correlate with improved outcomes among patients with CUP [15]. The current study investigated the multi‐omic landscape and clinical course of patients with CUP treated under the auspices of our precision medicine programme that leverages genomic characteristics to assess treatment approaches, including when customized combinations are given in order to optimize the degree of matching. In addition to blood‐ and tissue‐based next‐generation sequencing (NGS), multiple immune markers including, but not limited to, numerous checkpoints, inflammatory markers, myeloid suppression markers, metabolic immune escape markers and T‐cell priming markers were evaluated. Herein, we demonstrate that CUP patients frequently have actionable genomic alterations with high levels of evidence [per OncoKB database: (https://www.oncokb.org/)] [16] and that clinical outcomes were better in patients with high vs. low degrees of precision matching.

## 2 Materials and methods

### 2.1 Patients

We investigated clinical characteristics and treatment outcomes among patients with CUP at the University of California, San Diego (UCSD) Center for Personalized Cancer Therapy at Moores Cancer Center (*n* = 97). This study was performed in accordance with the guidelines of the UCSD Internal Review Board [PREDICT (Profile Related Evidence Determining Individualized Cancer Therapy) protocol; NCT02478931], and any investigational studies for which patients gave consent. Experiments were undertaken with the understanding and written consent of each subject. Data were gathered retrospectively on patients. Therapeutic determination was per recommendations in a face‐to‐face or electronic Molecular Tumor Board or by physician choice [17]. The goal of the Molecular Tumor Board recommendations was to optimize the degree of matching. The study methodologies conformed to the standards set by the Declaration of Helsinki.

### 2.2 Target identification through NGS, RNA and protein analysis

When available, we performed NGS on both tissue and plasma cfDNA to seek actionable genomic alterations. RNA and protein markers were also analysed as appropriate. For NGS, only deleterious alterations were evaluated (no variants of unknown significance were included in analyses).

The majority of tissue NGS was performed at Foundation Medicine (*N* = 61), which is a laboratory certified by Clinical Laboratory Improvement Amendments (CLIA; 182–315 genes; Cambridge, MA, USA, www.foundationmedicine.com) [18]. This method of sequencing allows for detection of copy‐number alterations, gene rearrangements, and somatic mutations with 99% specificity and > 99% sensitivity for base substitutions at ≥ 5 mutant allele frequency and > 95% sensitivity for copy‐number alterations. A threshold of ≥ 8 copies for gene amplification was used. A smaller subset of patients had tissue NGS done using other platforms, including UCSD (*N* = 2, 397 genes), Tempus, Inc. (*N* = 7, 595 genes; Chicago, IL, USA, www.tempus.com), Caris (*N* = 1, Irving, TX, USA, www.carislifesciences.com) and Human Longevity, Inc. (*N* = 1, San Diego, CA, USA, www.humanlongevity.com).

Blood‐derived cfDNA analysis was performed by Guardant Health (*N* = 66; Redwood City, CA, USA, www.guardanthealth.com), a CLIA‐certified laboratory with assay panels of 54 (*N* = 1), 68 (*N* = 4), 70 (*N* = 17) and 73 genes (*N* = 44) [19]. All cfDNA was sequenced, including somatic cfDNA and the germline cfDNA. Germline alterations were filtered out and not reported. The assay reports single nucleotide variants in all genes and selected copy‐number amplifications, fusions and indel events. In addition, blood‐derived cfDNA analysis was also performed by Tempus (*N* = 2) and Foundation Medicine (*N* = 4) in a small number of individuals.

Most PD‐L1 (programmed death‐ligand 1) immunohistochemistry (IHC) was performed at Foundation Medicine (https://www.foundationmedicine.com/; *N* = 36) followed by Tempus (*N* = 11; https://www.tempus.com/), OmniSeq (*N* = 6; https://www.omniseq.com/), Integrated Oncology (*N* = 1; https://www.integratedoncology.com/) and UCSD (*N* = 1). Antibodies for PD‐L1 IHC were Dako 22C3 (*N* = 30), SP142 (*N* = 22), Dako 28–8 (*N* = 1) and unspecified (*N* = 2). Tumour mutation burden was analysed in 64 patients by Foundation Medicine (*N* = 54), Tempus (*N* = 8), UCSD (*N* = 1) and Caris (*N* = 1).

Selected patients (*N* = 12) were evaluated for immune profiling markers with RNA sequencing by OmniSeq (www.omniseq.com; 51 immune markers) [20].

### 2.3 Matching Score to evaluate the degree of matching of tumours to therapy

We compared differences in outcomes according to a previously described molecular Matching Score [15, 21, 22]. Briefly, Matching Score was roughly defined as the number of alterations (not counting variants of unknown significance, VUS) targeted by administered drugs divided by the total number of pathogenic alterations (not counting VUSs) discerned. We did not differentiate between potential driver vs. passenger alterations. The higher the score (range, 0–100%), the better the match. For example, if a tumour harboured 10 deleterious genomic alterations and the patient was given two agents that targeted three of these alterations, the score would be calculated as 30% (3 of 10). Investigators who calculated the scores were blinded to participant outcomes. Because there can be heterogeneity between blood and tissue samples or among tissue biopsies, if a patient had ≥ 2 genomic test results, the pathogenic alterations in each test result were counted.

Other considerations were also relevant: (a) if a participant had ≥ 2 genomic aberrations that were in the same gene and potentially impacted the same signalling pathway, these abnormalities were counted as one; (b) if two agents simultaneously impacted the same anomaly in a well‐established synergistic manner (e.g. for *BRAF* aberrations, the Food and Drug Administration (FDA) approved combinations of dabrafenib (BRAF inhibitor) and trametinib (MEK inhibitor); for *ERBB2* aberrations, pertuzumab and trastuzumab antibodies), the impact was counted twice in both the numerator and denominator; (c) two pathogenic alterations in the same gene that potentially had different oncogenic impacts or were structurally distinct (e.g. amplification and mutation) were calculated as two; and (d) oestrogen or androgen receptor IHC positivity targeted by a hormone modulator (e.g. letrozole or enzalutamide) was also tallied as one in both the numerator and the denominator.

Antibodies were considered matched if their target was the product of the molecular aberration. For small molecule inhibitors, matching was based on low inhibitory concentration 50% (IC_50_) of the drug for the target (usually < 100 nm) or for protein effectors directly downstream of the anomalous gene product. Patients whose cancers harboured a *BRCA‐*related gene mutation were designated as matched if they were given PARP inhibitors or platinum cytotoxics. If an individual was given immune checkpoint blockade, the score was assigned as 100 per cent for results of microsatellite instability‐high (MSI‐H) or high tumour mutational burden (TMB‐H; ≥ 20 mutations·Mb^−1^) or high PD‐L1 IHC expression; the score was 50 per cent for results of TMB‐intermediate or low positive IHC PD‐L1. Individuals in the checkpoint blockade‐treated group who had, as an example, TMB‐intermediate (6–19 mutations·Mb^−1^) and were scored at 50 per cent, and also received matched targeted compounds, had the total score calculated as 50% + (X%/2) [X%/2 was defined as (number of alterations targeted by compounds given)/(total number of alterations X 2)]. For example, if a malignancy had intermediate TMB and the participant was given checkpoint blockade, but also had a *KIT* and *EGFR* alteration and received a *KIT* inhibitor in addition to immune checkpoint blockade, the score was 50 + 25% = 75%. We also counted *TP53* abnormalities as matched to small molecule inhibitors and antibodies with anti‐VEGF/VEGFR activity because several reports have stated that *TP53* tumour suppressor mutations are associated with enhanced VEGFA expression and that VEGF/VEGFR suppressive therapies correlated with improved therapy outcomes in patients with *TP53*‐aberrant cancer [23, 24, 25]. No match was scored as > 100%. More details on Matching Score calculations are in our prior publications [15, 21]. Any transcript or protein biomarker that was matched to a participant was counted as one in both the numerator and the denominator. We stratified patients according to Matching Scores 0–50% vs. > 50% (with unmatched patients scored as 0%). The cut‐off score of 50% was determined as reasonable for estimating the maximum SD ≥ 6 months with PR/CR rate in the higher score group using the receiver operating characteristic curve (ROC).

### 2.4 Endpoints and statistical methods

Patient demographic characteristics and biomarkers were summarized by descriptive statistics. Therapy response was evaluated by imaging (e.g. computed tomography and/or magnetic resonance imaging) and categorized into SD, PR, CR and progressive disease (PD) according to the treating physician's assessment. PFS was defined as time between treatment initiation and disease progression confirmed by clinical or imaging findings. OS was defined as time between initiation of treatment and last follow‐up. Patients still on treatment without progression at the last follow‐up date were censored for PFS at that date; patients alive at last follow‐up were censored for OS. (The last follow‐up event could be death.) The last data analysis date was 14 May 2019. Cox regression and log‐rank test were used to compare patient subgroups. Variables with *P* < 0.1 were included for multivariate analysis. *P*‐values ≤ 0.05 were considered significant. All tests were two‐sided. Statistical analyses were performed with help from co‐author RO utilizing spss version 24 software (IBM Corporation, Armonk, NY, USA).

## 3 Results

### 3.1 Demographic characteristics

We examined 6497 patients who were in the PREDICT database and found 97 patients with CUP (Fig. S1). Of these 97 patients, 62 were treated and evaluable for PFS and OS; 55 were treated and were evaluable for response; all evaluable patients had molecular diagnostic testing.

In this study, 59 of the 97 patients were women, and the median age at diagnosis was 63 years (range, 21–95 years; Table 1). Adenocarcinoma was the most common histology, but a variety of others were seen, including but not limited to neuroendocrine and squamous cell. All patients had advanced disease.

**Table 1:** Characteristics of 97 patients with carcinoma of unknown primary. cfDNA, cell‐free DNA; IHC, immunohistochemistry; MSI, microsatellite instability; NGS, next‐generation sequencing; TMB, tumour mutation burden. See Fig. S2 that describes overlap of test types.

Basic characteristics	
Age at diagnosis, median (range), year	63 (21–95)
Woman, number (%)	59 (60.8%)
Histology, number (%)	
Adenocarcinoma	52 (54%)
Neuroendocrine carcinoma	18 (19%)
Squamous cell carcinoma	10 (10%)
Poorly differentiated^a^	14 (14%)
Other^b^	3 (3%)
Molecular profiling	
Number of patients who had tissue NGS, number (%)	74 (76%)
Median number of characterized alterations from tissue NGS (range)	4 (0–25)
Number of patients who had cfDNA, number (%)	72 (74%)
Median number of characterized alterations from cfDNA (range)	2 (0–9)
Immune profiling	
Number of patients who had MSI testing	55 (*N* = 52 from tissue, *N* = 3 from cell‐free DNA)
Patients with MSI‐high/defect in a mismatch repair gene (%)	2/55 (3.6%)
Number of patients who had tissue‐based TMB testing	64
TMB‐Low (≤ 5 mutations·Mb^−1^)^c^	40/64 (62.5%)
TMB‐intermediate (6–19 mutations·Mb^−1^)^c^	18/64 (28.1%)
TMB‐high (≥ 20 mutations·Mb^−1^)^c^	6/64 (9.4%)
Patients with TMB ≥ 10 mutations·Mb^−1^	15/64 (23%)
Patients with *NTRK* fusion‐positive	None
Number of patients who had PD‐L1 testing by IHC	55
PD‐L1 positive^d^	17/55 (30.9%)
Number of patients who had comprehensive immune profiling by RNA sequencing	12

^a^ Not defined as adenocarcinoma, squamous cell carcinoma or neuroendocrine histology.

^b^ Sarcomatoid carcinoma, carcinoma (favoured but not limited to cholangiocarcinoma, hepatocellular carcinoma or breast primary), malignant epithelial neoplasm most consistent with gastrointestinal tract or biliary primary (*N* = 1 each).

^c^ Or as defined by the vendor/laboratory.

^d^ Defined as ≥ 1% by IHC from tumour (*N* = 16) or from tumour‐infiltrating lymphocyte (*N* = 1).

All patients had NGS performed on tissue and/or blood‐derived cfDNA. The median (range) number of deleterious alterations in tissue was 4 (range, 0–25); in cfDNA, 2 (0–9). Overall, based on the number of patients tested for the parameter, 3.6% (2/55) of tumours showed MSI‐H/defect in a mismatch repair gene; 9.4% (6/64) had TMB‐H (≥ 20 mutations·Mb^−1^); 23% (15/64) had TMB ≥ 10 mutations·Mb^−1^; and none of our patients had an *NTRK* fusion.

### 3.2 Genomic alterations as determined by NGS show complex molecular portfolios that differ between patients

**Fig. 1:**
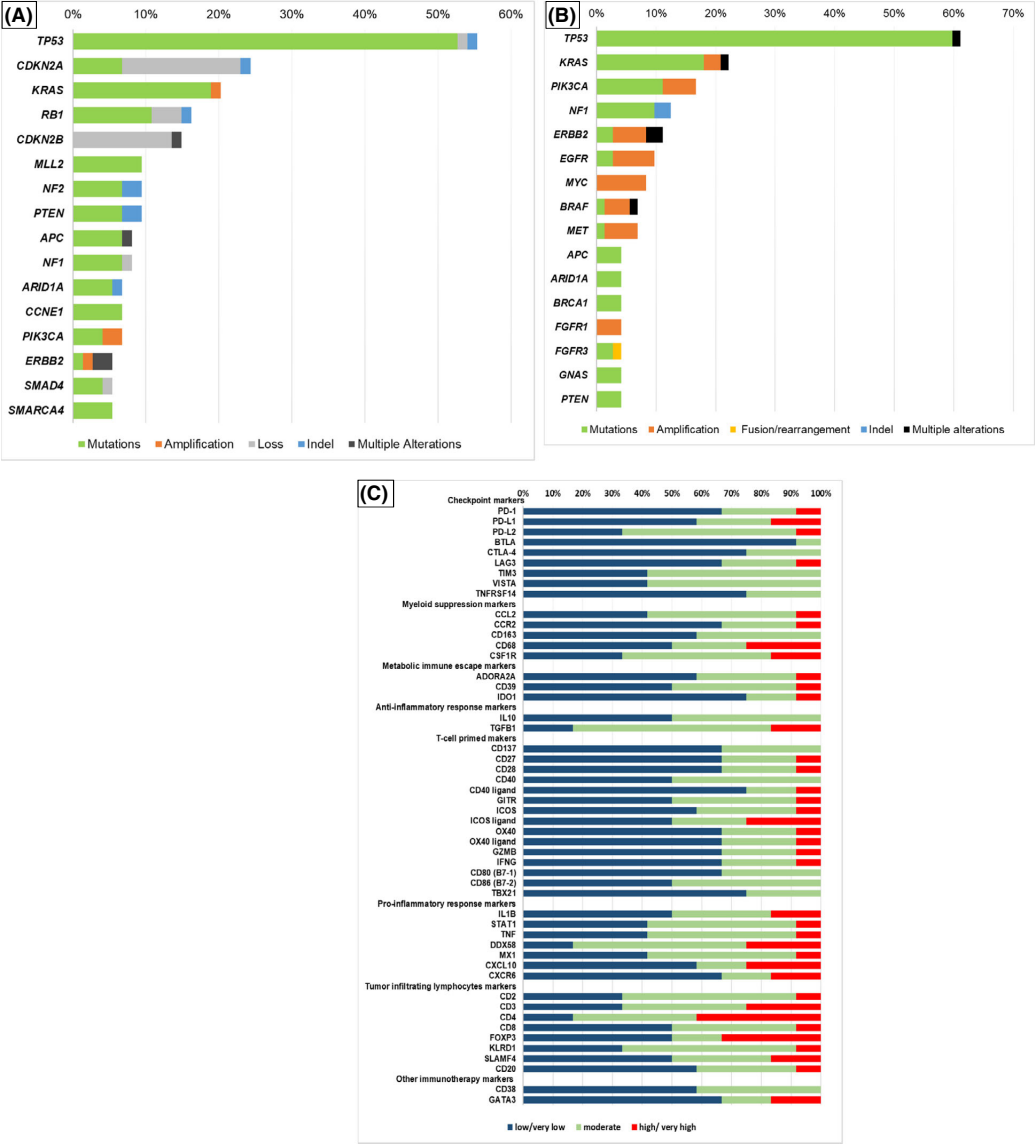
Pathogenic alterations in tissue NGS analysis (*N* = 74), blood‐derived cfDNA analysis (*N* = 72) and immune profiling markers with RNA sequencing (*N* = 12) among patients with CUP. (A) Pathogenic alterations in tissue NGS analysis among patients with CUP patients (*N* = 74). Numbers shown are percent of patients with the specified alteration. Included the genes with frequency of > 5%. See Table S1 for complete list of genomic alterations found by tissue NGS; percentage represents per cent of patient with alteration. (B) Pathogenic alterations in blood‐derived cfDNA analysis among patients with CUP (*N* = 72). Numbers shown are per cent of patients with the specified alteration. Included the genes with frequency of > 4%. See Table S2 for complete list of genomic alterations found by cfDNA assay; Percentage represents per cent of patient with alteration. (C) Immune profiling markers with RNA sequencing (OmniSeq) among patients with CUP (*N* = 12). Percentage represents per cent of patient with indicated expression. See also Table S3.

The median number of pathogenic alterations of the 74 patients who had tissue NGS testing was four (range, 0–25). Overall, 55.4% of patients who had tissue NGS profiling had a deleterious *TP53* alteration. Other prevalent tissue NGS deleterious alterations included *CDKN2A* (24.3%) and *KRAS* (20.3%; Fig. 1A and Table S1).

The median number of deleterious alterations in the 72 patients who had blood‐derived cfDNA testing was two (range, 0–9). The most common deleterious alterations in cfDNA were in *TP53* (61.1% of patients) followed by *KRAS* (20.8%) and *PIK3CA* (16.7%) (Fig. 1B and Table S2). No two patients had an identical molecular landscape, either on the tissue level or in the blood‐based cfDNA analysis.

### 3.3 Immune profiling: PD‐L1 testing by IHC and RNA expression of immune markers shows complex and heterogeneous CUP immunograms

Of the 55 patients who had PD‐L1 testing, 17 (30.9%) were PD‐L1‐positive on either tumour or immune cells (Table 1). Among the 12 patients who had tumour immune profiling by RNA expression (OmniSeq), each cancer's immune portfolio was different (Fig. 1C and Table S3). High levels of checkpoints PD‐1, PD‐L1, PD‐L2 and LAG‐3 were found in one to two tumours each; moderate levels of these checkpoints were found in additional cancers. Further, a subset of patients' malignancies had moderate levels of other checkpoint‐related markers, including but not limited to CTLA‐4, TIM3 and VISTA. More than one tumour had high/very high levels of the myeloid suppression markers CSF1R and CD68. Metabolic immune escape markers ADORA2, CD39 and IDO1 were each highly elevated in one tumour. TGFB1 was highly elevated in two tumours. ICOS ligand was the T‐cell‐primed marker most frequently highly expressed (*N* = 3 cancers). In addition to tumours with high/very high RNA levels of certain immune markers, almost each of the immune markers was at least intermediately elevated in 50% or more of tumours, though the pattern of immune marker change was highly heterogeneous between tumours.

### 3.4 CUP tumours frequently showed potentially actionable genomic alterations

Overall, 91% of patients (out of a total *N* = 97) had ≥ 1 deleterious genomic alterations in either tissue DNA or blood‐derived cfDNA as determined by NGS. Furthermore, treatment actionability as tabulated by a database that lists genomic alterations by their levels of evidence (OncoKB, https://www.oncokb.org/) [16] was evaluated: 54 of 97 patients had ≥ 1 Level 1, 2 or resistance (R1) alterations per OncoKB (most commonly the *KRAS* resistance marker; Tables S4 and S5). Furthermore, 30 of 97 patients had a genomic marker with Level 1 evidence (highest OncoKB level).

### 3.5 High degrees of matching of therapy to omic aberrations correlated with better outcome

Among 97 patients with CUP, 62 patients were evaluable for PFS and OS outcomes, and 55 patients were assessable for response to systemic therapy. Age, gender, histology and the number of prior lines of therapy had no correlation with clinical outcome (Tables 2 and 3). Patients who received immunotherapy (checkpoint blockade) vs. those who did not showed an increase in rate of SD ≥ 6 months/PR/CR (56% vs. 27%; *P* = 0.043), but multivariate *P*‐value was nonsignificant (*P* = 0.73); similarly, PFS showed a trend to improvement in univariate analysis (6.2 vs. 3.7 months; *P* = 0.063) but was nonsignificant in multivariate analysis; OS was not significantly different in patients who did and did not receive immune checkpoint blockade.

**Table 2:** Factors predictive for progression‐free survival and overall survival among patients with carcinomas of unknown primary who received systemic therapy (*N* = 62). CI, confidence interval; HR, hazard ratio.

Characteristics	Progression‐free survival				Overall survival			
	Univariate		Multivariate^a^		Univariate		Multivariate^a^	
	Median, months	*P*‐value	HR (95%CI)	*P*‐value	Median, months	*P*‐value	HR (95%CI)	*P*‐value
Age, years^b^								
≥ 64 [*N* = 30] vs. < 64 [*N* = 32]	3.8 vs. 2.8	0.09	0.81 (0.43–1.52)	0.51	6.9 vs. 10.8	0.89	—	—
Sex								
Female [*N* = 35] vs. male [*N* = 27]	4.0 vs. 2.7	0.47	—	—	6.9 vs. 11.9	0.35	—	—
Histology								
Adenocarcinoma [*N* = 32] vs. not [*N* = 30]	4.0 vs. 2.5	0.53	—	—	7.7 vs. 10.8	0.65	—	—
Treatment								
Administered as 1st line [*N* = 41] vs. ≥ 2nd line [*N* = 21]	3.1 vs. 3.7	0.80	—	—	7.7 vs. 11.7	0.63	—	—
≥ 2 drugs [*N* = 50] vs. single drug [*N* = 21]	3.1 vs. 3.8	0.95	—	—	10.8 vs. 20.7	0.60	—	—
Matched [*N* = 37] vs. Unmatched [*N* = 25]	3.7 vs. 3.8	0.41	—	—	13.4 vs. 6.9	0.31	—	—
Matching Score > 50% [*N* = 15] vs. ≤ 50% [*N* = 47]^c^	10.4 vs. 2.8	0.002	0.23 (0.07–0.74)	0.014	15.8 vs. 6.9	0.09	—	—
Immunotherapy based [*N* = 18] vs. other [*N* = 44]^d^	6.2 vs. 3.7	0.063	1.32 (0.54–3.22)	0.54	15.8 vs. 7.6	0.30	—	—

^a^ Variables with *P*‐values < 0.1 in the univariate analysis were included for multivariate analysis. Multivariate analysis was not performed for the overall survival since Matching Score was the only variable with *P* < 0.1.

^b^ Age at treatment initiation. Dichotomized by the median value.

^c^ Low Matching Score group includes 25 patients with Matching Score = 0 (unmatched) and 22 patients with Matching Score of 1 to 50%.

^d^ *N* = 44 patients received non‐immunotherapy‐based therapies.

**Table 3:** Factors predictive for clinical benefit (SD ≥ 6 months/PR/CR) with systemic therapy among patients with carcinomas of unknown primary (*N* = 55). CI, confidence interval; OR, odds ratio.

Characteristics	Treatment response (SD ≥ 6 months/PR/CR) (*N* = 55)^a^			
	Univariate		Multivariate	
	Rate (*N*)	*P*‐value	OR (95%CI)	*P*‐value
Age, years^b^				
≥ 64 [*N* = 28] vs. < 64 [*N* = 27]	43% (12/28) vs. 30% (8/27)	0.40	—	—
Sex				
Female [*N* = 30] vs. male [*N* = 25]	40% (12/30) vs. 32% (8/25)	0.59	—	—
Histology				
Adenocarcinoma [*N* = 29] vs. not [*N* = 26]	45% (13/29) vs. 27% (7/26)	0.26	—	—
Treatment				
Administered as 1st line [*N* = 36] vs. ≥ 2nd line [*N* = 19]	36% (13/36) vs. 37% (7/19)	> 0.99	—	—
≥ 2 drugs [*N* = 45] vs. single drug [*N* = 10]	40% (18/45) vs. 20% (2/10)	0.30	—	—
Matched [*N* = 34] vs. unmatched [*N* = 21]	44% (15/34) vs. 34% (5/21)	0.16	—	—
Matching Score > 50% [*N* = 14] vs. ≤ 50 [*N* = 41]^c^	71% (10/14) vs. 24% (10/41)	0.003	6.67 (1.30–33.3)	0.022
Immunotherapy based [*N* = 18] vs. other [*N* = 37]^d^	56% (10/18) vs. 27% (10/37)	0.043	1.32 (0.29–5.88)	0.73

^a^ Excluded 6 patients who had ongoing SD less than 6 months at the time of data cut‐off. Additionally, one patient was not evaluable for response assessment.

^b^ Age at treatment initiation. Dichotomized by the median value.

^c^ Low Matching Score group includes 21 patients with Matching Score = 0 (unmatched) and 20 patients with Matching Score of 1 to 50.

^d^ N = 37 patients received non‐immunotherapy‐based therapies.

**Fig. 2:**
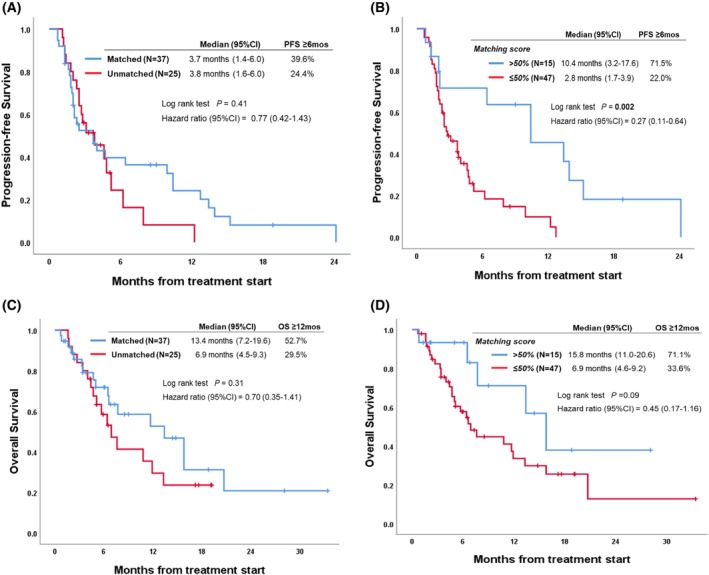
Progression‐free survival and overall survival depending on matched vs. unmatched therapy and the Matching Score of treatment (*N* = 62). (A) Progression‐free survival depending on whether patients received matched vs. unmatched therapy (*N* = 62). Patients whose tumour therapy matched to their alterations vs. those whose tumours were unmatched therapeutically to their alterations showed no difference in progression‐free survival (matched vs. unmatched: 3.7 vs. 3.8 months, *P* = 0.41, univariate). (B) Progression‐free survival depending on the Matching Score of treatment (see Materials and methods for definition of Matching Score) (*N* = 62). Patients who received therapy with high degree of matching (Matching Score > 50%) had significantly better progression‐free survival when compared to patients treated with low degree of matching (Matching Score ≤ 50%; median progression‐free survival: 10.4 vs. 2.8 months, *P* = 0.002, univariate). (C) Overall survival depending on whether patients received matched vs. unmatched therapy (*N* = 62). Patients whose tumour therapy matched to their alterations vs. those whose tumours were unmatched therapeutically to their alterations showed no difference in overall survival (matched vs. unmatched: 13.4 vs. 6.9 months, *P* = 0.31, univariate). (D) Overall survival depending on the Matching Score of treatment (see Materials and methods for definition of Matching Score; *N* = 62). Patients who received therapy with high degree of matching (Matching Score > 50%) had trend toward better overall survival when compared to patients treated with low degree of matching (Matching Score ≤ 50%; median overall survival: 15.8 vs. 6.9 months, *P* = 0.09, univariate).

**Fig. 3:**
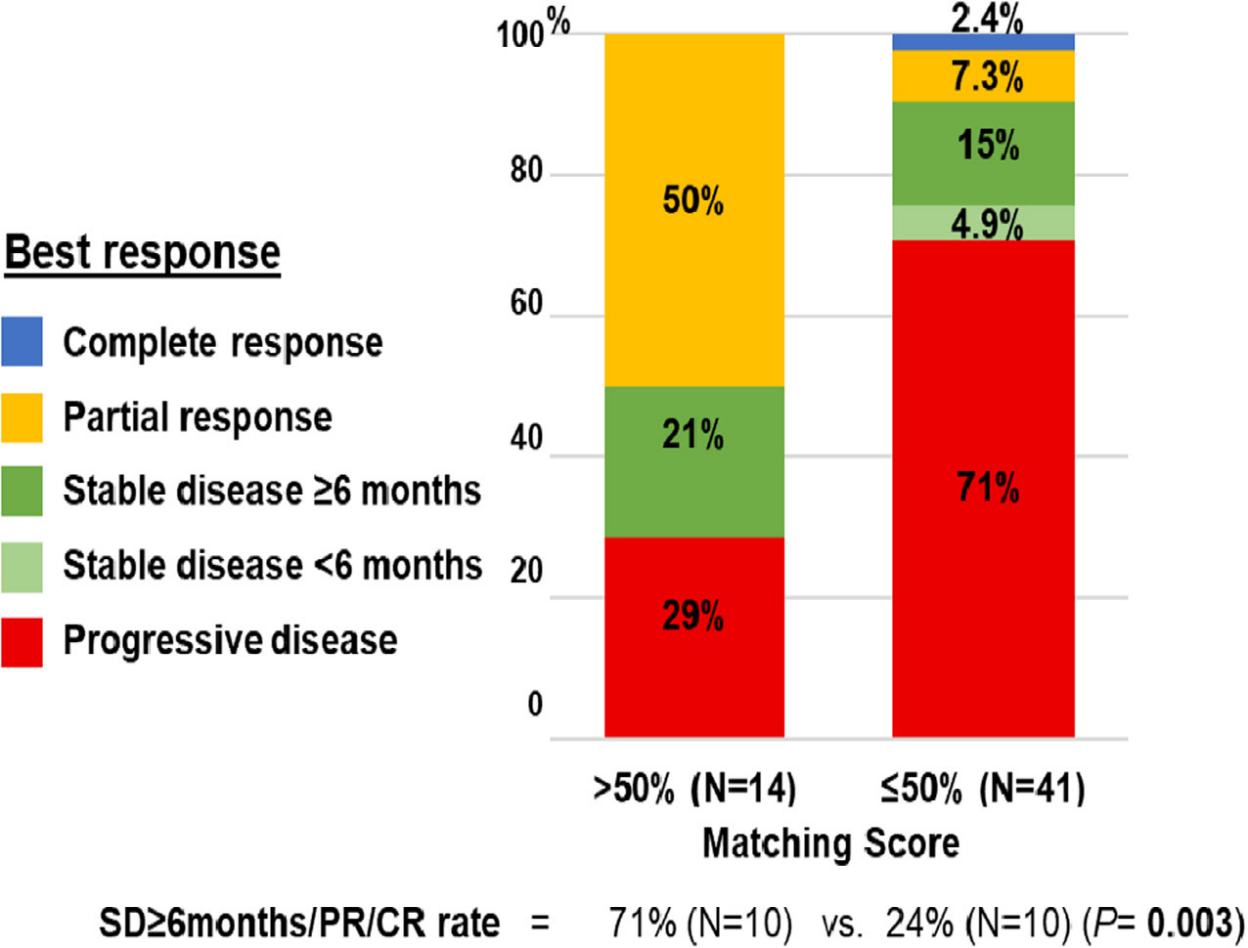
Treatment benefit/response according to Matching Score among patients with carcinomas of unknown primary (*N* = 55)*. Patients who received therapy with high Matching Score (> 50%) showed significant improvement in SD ≥ 6 months/PR/CR when compared to patients who had therapy with low Matching Score (≤ 50%; 71% vs. 24%, *P* = 0.003, univariate).* Excluded six patients who had ongoing SD < 6 months at the time of data cut‐off. One patient was not evaluable for response assessment. CR, complete response; PD, progressive disease; PR, partial response; SD, stable disease.

We also explored the relationship between the degree to which therapy matched tumour alterations and outcome (Tables 2 and 3; Figs 2 and 3). Examining patients whose tumour therapy matched to their alterations vs. those whose tumours were unmatched therapeutically to their alterations showed no difference in any outcome parameters (Tables 2 and 3; Fig. 2A,C). The number of drugs given to a patient also did not correlate with outcome. However, examining patients with high vs. low degrees of matching (Matching Score > 50% vs. ≤ 50%) showed significant differences (Figs 2B,D and 3, Table S6): SD ≥ 6 months/PR/CR, 71% vs. 24% (univariate *P* = 0.003; multivariate *P* = 0.022); median PFS, 10.4 vs. 2.8 (univariate *P* = 0.002; multivariate *P* = 0.014); and median OS, 15.8 vs. 6.9 [*P* = 0.09 (trend, univariate only); Tables 2 and 3]. Therefore, the only variable that independently correlated with improvement in any outcome parameter was the degree to which there was matching of tumour alterations to therapy.

## 4 Discussion

Carcinoma of unknown primary is an uncommon malignancy, afflicting less than 5% of patients with cancer, with a poor prognosis. By definition, the primary cancer diagnosis is unknown [26, 27]. Assays have been created to establish site of origin [28], but these assays have not effectively navigated patients to treatments that improve outcomes [7, 8]. Recently, with the dawn of the precision medicine era, clinical‐grade tissue and blood‐based NGS have become accessible, as has multiple other biomarker testing at the transcriptomic and proteomic level [29]. Among 6497 who were enrolled in the PREDICT study at our Center for Personalized Cancer Therapy, we found 97 with CUP, of whom 62 were evaluable for therapeutic outcome.

Patients had a variety of potential important biomarkers. Indeed, there were only 9 of 97 patients (9.3%) who had no deleterious genomic alterations in either tissue DNA or blood cfDNA NGS. Moreover, we explored the landscape of alterations based on their druggability, as determined by OncoKB, a database that consists of a curated list of somatic molecular alterations defined by their level of evidence (https://www.oncokb.org/) [16]; 56% (of 97 patients) had ≥ 1 actionable marker per OncoKB (Tables S4 and S5); 93% had ≥ 1 potentially actionable marker per UCSD (latter includes PD‐L1 IHC positive) [12, 30]; and 31% of patients had a marker with the highest level of evidence (Level 1) by OncoKB. The median number of deleterious genomic alterations in tissue DNA was 4 (range, 0–25); in blood‐derived cfDNA, 2 (0–9). Aberrations in *TP5*3 and *KRAS* genes were among the most frequent deleterious alterations in tissue DNA and in blood‐derived cfDNA. Recently, the FDA has approved several drugs for tissue‐agnostic use: 3.6% of our tested patients had MSI‐H/defect in a mismatch repair gene, and 23% had TMB ≥ 10 mutations·Mb^−1^ (approved for the checkpoint inhibitor pembrolizumab); [31, 32] no patient had an *NTRK* fusion (approved for the NTRK inhibitors larotrectinib or entrectinib) [33, 34]. The latter may not be a surprise, since *NTRK* fusions occur in only 0.3% of patients with diverse advanced cancers [35]. Overall, 30.9% of tumours were positive for PD‐L1 by IHC, an approved biomarker for immune checkpoint blockade [36]. Twelve patients also had in‐depth immune interrogation of their tumour. A variety of theoretically druggable targets were found including, but not limited to, LAG‐3, CSF1R, ADORA2 and IDO1, all of which can be impacted by compounds that are approved or in clinical trials. Both NGS and immune profiling revealed that CUPs are complex and heterogeneous and that they differ from each other at both the mutanome and the immunome level.

When we explored clinical outcomes, important themes emerged. Classic variables such as age, gender, histology (adenocarcinoma vs. squamous vs. neuroendocrine vs. other), number of prior lines of therapy, the number of drugs given in the treatment and administration of immune checkpoint blockade had no significant association with outcome in multivariate analysis (Tables 2 and 3). However, the patients with partial response received immunotherapy (anti‐PD1; based on PDL1 positive or high TMB), though in all cases but one, together with another agent. Furthermore, determining whether or not patients' tumours were matched or unmatched to therapy was not correlated with any outcome parameter. In contrast, patients with high vs. low degrees of matching (as reflected by Matching Score > 50% vs. ≤ 50%) showed significant differences in rates of clinical benefit (SD ≥ 6 months/PR/CR) and PFS in multivariate analysis and a trend toward better survival. Importantly, the degree of matching of tumours to therapy was the only variable that independently correlated with improvement in any outcome parameter. These data indicate that it is critical to recognize that simply matching targeted therapeutics to genomic aberrations was not sufficient to generate improved patient outcomes. The findings in our patients with CUP are consistent with previously reported data showing that high vs. low degrees of matching are significantly associated with higher response rates and longer PFS and OS across advanced cancers [15, 21].

There are several important limitations to the current study. The number of treated patients is small, and the analysis was performed retrospectively. Further, only a subgroup of patient tumours had comprehensive immune profiling. Although most patients were discussed in a Molecular Tumor Board, the therapy was not scripted. The latter permitted flexibility for the physicians to choose the best management for each patient, based on their medical and personal circumstances, was akin to real‐world practice. Biases related to differing prognoses or differing NGS panels may also be present. In the future, prospective randomized studies are needed to better validate our findings, particularly, the utility of the Matching Score. Further investigation is also required to discern whether certain combinations of targeted therapies are more efficacious than others in patients with the same genomic findings. Finally, the results are hypothesis‐generating as the scoring system requires external validation.

## 5 Conclusion

In summary, among patients with CUP, the majority had actionable genomic alterations as determined by NGS on tissue DNA or blood‐derived cfDNA. Furthermore, 3.6% of cancers had MSI‐H/defect in mismatch repair gene and 23% had TMB ≥ 10 mutations·Mb^−1^, each of which is FDA‐approved tissue‐agnostic genomic biomarkers for immunotherapy. In addition, 30.9% of CUPs were PD‐L1‐positive by IHC, also an FDA‐approved biomarker for checkpoint blockade. No two patients had the same portfolio of molecular alterations or of immune profile perturbations. Furthermore, most patients had several genomic alterations and, for those tested, multiple immune portfolio changes, speaking to the need for NGS and comprehensive immune interrogation in order to inform individualized combination treatment. Consistent with this observation, the degree to which CUPs were matched to a tailored treatment combination was the only factor that was independently associated with improved outcome parameters. These data are consistent with other prior trials, suggesting that combination therapy with high degrees of matching may be effective, even where matched monotherapies are not active [15, 21, 37]. Larger prospective trials of an N‐of‐One matching strategy for CUPs informed by genomic and immune profiling are warranted.

## Conflict of interest

Shumei Kato serves as a consultant for Foundation Medicine; and has received speaker's fees from Roche and research grant from ACT Genomics, Sysmex, Konica Minolta and OmniSeq. Sophia Gumas, Jacob Adashek, Ryosuke Okamura and Suzanna Lee have no disclosures. Jason Sicklick receives research funding from Novartis Pharmaceuticals, Amgen Pharmaceuticals and Foundation Medicine; consultant fees from Grand Rounds, Loxo and Deciphera; and speaker's fees from Roche. Razelle Kurzrock receives stock and other equity interests from IDbyDNA, CureMatch and CureMetrix; consulting or advisory role fees from Gaido, TD2, Bicara, Turning Point, X‐Biotech, Actuate Therapeutics, Roche, NeoMed, Soluventis and Pfizer; speaker's fee from Roche; and research funding from Incyte, Genentech, Merck Serono, Pfizer, Sequenom, Foundation Medicine, Guardant Health, Grifols, Konica Minolta, DeBiopharm, Boehringer Ingelheim and OmniSeq [all institutional]; and serves as a board member for CureMatch, Inc and CureMetrix Inc.

## Author contributions

SK and RK conceived and designed the project. SG, RO and SL acquired the data. SK, SG, JJA, JKS and RK analysed and interpreted the data. SK, JJA, JKS and RK drafted versions of the manuscript. All authors reviewed and approved the final draft of the manuscript.

### Peer review

The peer review history for this article is available at https://publons.com/publon/10.1002/1878‐0261.13293.

## Supporting information


**Table S1.** Tissue NGS genomic alterations in patients with carcinoma of unknown primary (*N* = 74).
**Table S2**. Blood‐derived cfDNA analysis in patients with carcinoma of unknown primary (*N* = 72).
**Table S3**. Immune profiling with RNA sequencing in patients with carcinoma of unknown primary (*N* = 12).
**Table S4.** OncoKB annotation and level of evidence (www.oncokb.org). (accessed as of July 17, 2020).
**Table S5.** Alterations with level of evidence according to OncoKB annotation.
**Table S6**. Details of the 7 patients with high Matching Score (> 50) who achieved a partial response.
**Fig. S1.** CONSORT diagram of the study
**Fig. S2.** Venn diagram shows overlap of test types with further detail in the chart.

## Data Availability

Data are available upon reasonable request.

## References

[1] Pavlidis N , Pentheroudakis G . Cancer of unknown primary site. Lancet. 2012;379:1428–35. 10.1016/S0140-6736(11)61178-1 22414598

[2] Fizazi K , Greco FA , Pavlidis N , Pentheroudakis G , Group EGW . Cancers of unknown primary site: ESMO Clinical Practice Guidelines for diagnosis, treatment and follow‐up. Ann Oncol. 2011;22(Suppl 6):vi64–8. 10.1093/annonc/mdr389 21908507

[3] Massard C , Loriot Y , Fizazi K . Carcinomas of an unknown primary origin—diagnosis and treatment. Nat Rev Clin Oncol. 2011;8:701–10. 10.1038/nrclinonc.2011.158 22048624

[4] Varadhachary GR , Raber MN . Carcinoma of unknown primary site. N Engl J Med. 2014;371:2040. 10.1056/NEJMc1411384 25409386

[5] Briasoulis E , Kalofonos H , Bafaloukos D , Samantas E , Fountzilas G , Xiros N , et al. Carboplatin plus paclitaxel in unknown primary carcinoma: a phase II Hellenic Cooperative Oncology Group Study. J Clin Oncol. 2000;18:3101–7. 10.1200/JCO.2000.18.17.3101 10963638

[6] Greco FA , Erland JB , Morrissey LH , Burris HA 3rd , Hermann RC , Steis R , et al. Carcinoma of unknown primary site: phase II trials with docetaxel plus cisplatin or carboplatin. Ann Oncol. 2000;11:211–5. 10.1023/a:1008369812295 10761758

[7] Fizazi K , Maillard A , Penel N , Baciarello G , Allouache D , Daugaard G , et al. LBA15_PR A phase III trial of empiric chemotherapy with cisplatin and gemcitabine or systemic treatment tailored by molecular gene expression analysis in patients with carcinomas of an unknown primary (CUP) site (GEFCAPI 04). Ann Oncol. 2019;30:mdz394.

[8] Hayashi H , Kurata T , Takiguchi Y , Arai M , Takeda K , Akiyoshi K , et al. Randomized phase II trial comparing site‐specific treatment based on gene expression profiling with carboplatin and paclitaxel for patients with cancer of unknown primary site. J Clin Oncol. 2019;37:570–9. 10.1200/JCO.18.00771 30653423

[9] Naing A , Meric‐Bernstam F , Stephen B , Karp DD , Hajjar J , Rodon Ahnert J , et al. Phase 2 study of pembrolizumab in patients with advanced rare cancers. J Immunother Cancer. 2020;8:e000347. 10.1136/jitc-2019-000347 32188704 PMC7078933

[10] Gatalica Z , Millis SZ , Vranic S , Bender R , Basu GD , Voss A , et al. Comprehensive tumor profiling identifies numerous biomarkers of drug response in cancers of unknown primary site: analysis of 1806 cases. Oncotarget. 2014;5:12440–7. 10.18632/oncotarget.2574 25415047 PMC4322997

[11] Hayashi H , Takiguchi Y , Minami H , Akiyoshi K , Segawa Y , Ueda H , et al. Site‐specific and targeted therapy based on molecular profiling by next‐generation sequencing for cancer of unknown primary site: a nonrandomized phase 2 clinical trial. JAMA Oncol. 2020;6:1931–8. 10.1001/jamaoncol.2020.4643 33057591 PMC7563669

[12] Kato S , Krishnamurthy N , Banks KC , De P , Williams K , Williams C , et al. Utility of genomic analysis in circulating tumor DNA from patients with carcinoma of unknown primary. Cancer Res. 2017;77:4238–46. 10.1158/0008-5472.CAN-17-0628 28642281 PMC5729906

[13] Ross JS , Wang K , Gay L , Otto GA , White E , Iwanik K , et al. Comprehensive genomic profiling of carcinoma of unknown primary site: new routes to targeted therapies. JAMA Oncol. 2015;1:40–9. 10.1001/jamaoncol.2014.216 26182302

[14] Ross J , Sokol E , Moch H , Mileshkin L , Baciarello G , Losa F , et al. Comprehensive genomic profiling (CGP) of carcinoma of unknown primary origin (CUP): Retrospective molecular classification of potentially eligible patients (pts) for targeted or immunotherapy treatment (tx) using the prospective CUPISCO trial's criteria. Ann Oncol. 2019;30:v934.

[15] Sicklick JK , Kato S , Okamura R , Schwaederle M , Hahn ME , Williams CB , et al. Molecular profiling of cancer patients enables personalized combination therapy: the I‐PREDICT study. Nat Med. 2019;25:744–50. 10.1038/s41591-019-0407-5 31011206 PMC6553618

[16] Chakravarty D , Gao J , Phillips SM , Kundra R , Zhang H , Wang J , et al. OncoKB: a precision oncology knowledge base. JCO Precis Oncol. 2017;2017:1–16. 10.1200/PO.17.00011 PMC5586540 28890946

[17] Schwaederle M , Parker BA , Schwab RB , Daniels GA , Piccioni DE , Kesari S , et al. Precision oncology: the UC San Diego Moores Cancer Center PREDICT Experience. Mol Cancer Ther. 2016;15:743–52. 10.1158/1535-7163.MCT-15-0795 26873727

[18] Frampton GM , Fichtenholtz A , Otto GA , Wang K , Downing SR , He J , et al. Development and validation of a clinical cancer genomic profiling test based on massively parallel DNA sequencing. Nat Biotechnol. 2013;31:1023–31. 10.1038/nbt.2696 24142049 PMC5710001

[19] Lanman RB , Mortimer SA , Zill OA , Sebisanovic D , Lopez R , Blau S , et al. Analytical and clinical validation of a digital sequencing panel for quantitative, highly accurate evaluation of cell‐free circulating tumor DNA. PLoS ONE. 2015;10:e0140712. 10.1371/journal.pone.0140712 26474073 PMC4608804

[20] Kato S , Okamura R , Kumaki Y , Ikeda S , Nikanjam M , Eskander R , et al. Expression of TIM3/VISTA checkpoints and the CD68 macrophage‐associated marker correlates with anti‐PD1/PDL1 resistance: implications of immunogram heterogeneity. Onco Targets Ther. 2020;9:1708065. 10.1080/2162402X.2019.1708065 PMC7028323 32117584

[21] Kato S , Kim KH , Lim HJ , Boichard A , Nikanjam M , Weihe E , et al. Real‐world data from a molecular tumor board demonstrates improved outcomes with a precision N‐of‐One strategy. Nat Commun. 2020;11:4965. 10.1038/s41467-020-18613-3 33009371 PMC7532150

[22] Rodon J , Soria JC , Berger R , Miller WH , Rubin E , Kugel A , et al. Genomic and transcriptomic profiling expands precision cancer medicine: the WINTHER trial. Nat Med. 2019;25:751–8. 10.1038/s41591-019-0424-4 31011205 PMC6599610

[23] Koehler K , Liebner D , Chen JL . TP53 mutational status is predictive of pazopanib response in advanced sarcomas. Ann Oncol. 2016;27:539–43. 10.1093/annonc/mdv598 26646755 PMC5006122

[24] Said R , Hong DS , Warneke CL , Lee JJ , Wheler JJ , Janku F , et al. P53 mutations in advanced cancers: clinical characteristics, outcomes, and correlation between progression‐free survival and bevacizumab‐containing therapy. Oncotarget. 2013;4:705–14. 10.18632/oncotarget.974 23670029 PMC3742831

[25] Schwaederle M , Lazar V , Validire P , Hansson J , Lacroix L , Soria JC , et al. VEGF‐A expression correlates with TP53 mutations in non‐small cell lung cancer: implications for antiangiogenesis therapy. Cancer Res. 2015;75:1187–90. 10.1158/0008-5472.CAN-14-2305 25672981

[26] Kato S , Alsafar A , Walavalkar V , Hainsworth J , Kurzrock R . Cancer of unknown primary in the molecular era. Trends Cancer. 2021;7:465–77. 10.1016/j.trecan.2020.11.002 33516660 PMC8062281

[27] Lee MS , Sanoff HK . Cancer of unknown primary. BMJ. 2020;371:m4050. 10.1136/bmj.m4050 33288500

[28] Hainsworth JD , Rubin MS , Spigel DR , Boccia RV , Raby S , Quinn R , et al. Molecular gene expression profiling to predict the tissue of origin and direct site‐specific therapy in patients with carcinoma of unknown primary site: a prospective trial of the Sarah Cannon research institute. J Clin Oncol. 2013;31:217–23. 10.1200/JCO.2012.43.3755 23032625

[29] Subbiah V , Kurzrock R . Challenging standard‐of‐care paradigms in the precision oncology era. Trends Cancer. 2018;4:101–9. 10.1016/j.trecan.2017.12.004 29458960 PMC5822744

[30] Bieg‐Bourne CC , Millis SZ , Piccioni DE , Fanta PT , Goldberg ME , Chmielecki J , et al. Next‐generation sequencing in the clinical setting clarifies patient characteristics and potential actionability. Cancer Res. 2017;77:6313–20. 10.1158/0008-5472.CAN-17-1569 28939679 PMC5690871

[31] Lemery S , Keegan P , Pazdur R . First FDA approval agnostic of cancer site – when a biomarker defines the indication. N Engl J Med. 2017;377:1409–12. 10.1056/NEJMp1709968 29020592

[32] Subbiah V , Solit DB , Chan TA , Kurzrock R . The FDA approval of pembrolizumab for adult and pediatric patients with tumor mutational burden (TMB) >/=10: a decision centered on empowering patients and their physicians. Ann Oncol. 2020;31:1115–8. 10.1016/j.annonc.2020.07.002 32771306

[33] Doebele RC , Drilon A , Paz‐Ares L , Siena S , Shaw AT , Farago AF , et al. Entrectinib in patients with advanced or metastatic NTRK fusion‐positive solid tumours: integrated analysis of three phase 1‐2 trials. Lancet Oncol. 2020;21:271–82. 10.1016/S1470-2045(19)30691-6 31838007 PMC7461630

[34] Drilon A , Laetsch TW , Kummar S , DuBois SG , Lassen UN , Demetri GD , et al. Efficacy of larotrectinib in TRK fusion‐positive cancers in adults and children. N Engl J Med. 2018;378:731–9. 10.1056/NEJMoa1714448 29466156 PMC5857389

[35] Okamura R , Boichard A , Kato S , Sicklick JK , Bazhenova L , Kurzrock R . Analysis of NTRK alterations in pan‐cancer adult and pediatric malignancies: implications for NTRK‐targeted therapeutics. JCO Precis Oncol. 2018;2018:PO.18.00183. 10.1200/PO.18.00183 30637364 PMC6329466

[36] Patel SP , Kurzrock R . PD‐L1 expression as a predictive biomarker in cancer immunotherapy. Mol Cancer Ther. 2015;14:847–56. 10.1158/1535-7163.MCT-14-0983 25695955

[37] Kato S , Adashek JJ , Shaya J , Okamura R , Jimenez RE , Lee S , et al. Concomitant MEK and cyclin gene alterations: implications for response to targeted therapeutics. Clin Cancer Res. 2021;27:2792–7. 10.1158/1078-0432.CCR-20-3761 33472910 PMC11005753

