# Supplementary material for: Multi‐omic analysis in carcinoma of unknown primary (CUP): therapeutic impact of knowing the unknown
Source: Mol Oncol. 2023 Nov 16;18(4):956–68. doi: 10.1002/1878-0261.13293 (PMC10994241; doi:10.1002/1878-0261.13293)
Supplement: Supplementary file 1 — Table S1. Tissue NGS genomic alterations in patients with carcinoma of unknown primary (N = 74). Table S2. Blood‐derived cfDNA analysis in patients with carcinoma of unknown primary (N = 72). Table S3. Immune profiling with RNA sequencing in patients with carcinoma of unknown primary (N = 12). Table S4. OncoKB annotation and level of evidence (www.oncokb.org). (accessed as of July 17, 2020). Table S5. Alterations with level of evidence according to OncoKB annotation. Table S6. Details of the 7 patients with high Matching Score (> 50) who achieved a partial response. Fig. S1. CONSORT diagram of the study Fig. S2. Venn diagram shows overlap of test types with further detail in the chart. [file MOL2-18-956-s001.doc]

**Supplemental Table 1**. Tissue NGS genomic alterations in patients with carcinoma of unknown primary (N = 74).

|  | **Characterized alterations**  **No. (%)** | **Mutations**  **No. (%)** | **Amplification**  **No. (%)** | **Loss**  **No. (%)** | **Fusion/ Rearrangement**  **No. (%)** | **Indel**  **No. (%)** | **Multiple alterations**  **No. (%)** |
| --- | --- | --- | --- | --- | --- | --- | --- |
| ***TP53*** | 41 (55.4%) | 39 (52.7%) | 0 (0.0%) | 1 (1.4%) | 0 (0.0%) | 1 (1.4%) | 0 (0.0%) |
| ***CDKN2A*** | 18 (24.3%) | 5 (6.8%) | 0 (0.0%) | 12 (16.2%) | 0 (0.0%) | 1 (1.4%) | 0 (0.0%) |
| ***KRAS*** | 15 (20.3%) | 14 (18.9%) | 1 (1.4%) | 0 (0.0%) | 0 (0.0%) | 0 (0.0%) | 0 (0.0%) |
| ***RB1*** | 12 (16.2%) | 8 (10.8%) | 0 (0.0%) | 3 (4.1%) | 0 (0.0%) | 1 (1.4%) | 0 (0.0%) |
| ***CDKN2B*** | 11 (14.9%) | 0 (0.0%) | 0 (0.0%) | 10 (13.5%) | 0 (0.0%) | 0 (0.0%) | 1 (1.4%) |
| ***MLL2*** | 7 (9.5%) | 7 (9.5%) | 0 (0.0%) | 0 (0.0%) | 0 (0.0%) | 0 (0.0%) | 0 (0.0%) |
| ***NF2*** | 7 (9.5%) | 5 (6.8%) | 0 (0.0%) | 0 (0.0%) | 0 (0.0%) | 2 (2.7%) | 0 (0.0%) |
| ***PTEN*** | 7 (9.5%) | 5 (6.8%) | 0 (0.0%) | 0 (0.0%) | 0 (0.0%) | 2 (2.7%) | 0 (0.0%) |
| ***APC*** | 6 (8.1%) | 5 (6.8%) | 0 (0.0%) | 0 (0.0%) | 0 (0.0%) | 0 (0.0%) | 1 (1.4%) |
| ***NF1*** | 6 (8.1%) | 5 (6.8%) | 0 (0.0%) | 1 (1.4%) | 0 (0.0%) | 0 (0.0%) | 0 (0.0%) |
| ***ARID1A*** | 5 (6.8%) | 4 (5.4%) | 0 (0.0%) | 0 (0.0%) | 0 (0.0%) | 1 (1.4%) | 0 (0.0%) |
| ***CCNE1*** | 5 (6.8%) | 5 (6.8%) | 0 (0.0%) | 0 (0.0%) | 0 (0.0%) | 0 (0.0%) | 0 (0.0%) |
| ***PIK3CA*** | 5 (6.8%) | 3 (4.1%) | 2 (2.7%) | 0 (0.0%) | 0 (0.0%) | 0 (0.0%) | 0 (0.0%) |
| ***ERBB2*** | 4 (5.4%) | 1 (1.4%) | 1 (1.4%) | 0 (0.0%) | 0 (0.0%) | 0 (0.0%) | 2 (2.7%) |
| ***SMAD4*** | 4 (5.4%) | 3 (4.1%) | 0 (0.0%) | 1 (1.4%) | 0 (0.0%) | 0 (0.0%) | 0 (0.0%) |
| ***SMARCA4*** | 4 (5.4%) | 4 (5.4%) | 0 (0.0%) | 0 (0.0%) | 0 (0.0%) | 0 (0.0%) | 0 (0.0%) |
| ***BAP1*** | 3 (4.1%) | 3 (4.1%) | 0 (0.0%) | 0 (0.0%) | 0 (0.0%) | 0 (0.0%) | 0 (0.0%) |
| ***BCOR*** | 3 (4.1%) | 3 (4.1%) | 0 (0.0%) | 0 (0.0%) | 0 (0.0%) | 0 (0.0%) | 0 (0.0%) |
| ***BRAF*** | 3 (4.1%) | 3 (4.1%) | 0 (0.0%) | 0 (0.0%) | 0 (0.0%) | 0 (0.0%) | 0 (0.0%) |
| ***CTNNB1*** | 3 (4.1%) | 1 (1.4%) | 0 (0.0%) | 0 (0.0%) | 0 (0.0%) | 2 (2.7%) | 0 (0.0%) |
| ***EGFR*** | 3 (4.1%) | 0 (0.0%) | 3 (4.1%) | 0 (0.0%) | 0 (0.0%) | 0 (0.0%) | 0 (0.0%) |
| ***EP300*** | 3 (4.1%) | 3 (4.1%) | 0 (0.0%) | 0 (0.0%) | 0 (0.0%) | 0 (0.0%) | 0 (0.0%) |
| ***ERBB3*** | 3 (4.1%) | 1 (1.4%) | 2 (2.7%) | 0 (0.0%) | 0 (0.0%) | 0 (0.0%) | 0 (0.0%) |
| ***LRP1B*** | 3 (4.1%) | 2 (2.7%) | 0 (0.0%) | 0 (0.0%) | 0 (0.0%) | 1 (1.4%) | 0 (0.0%) |
| ***MET*** | 3 (4.1%) | 1 (1.4%) | 1 (1.4%) | 0 (0.0%) | 0 (0.0%) | 0 (0.0%) | 1 (1.4%) |
| ***MTAP*** | 3 (4.1%) | 0 (0.0%) | 0 (0.0%) | 3 (4.1%) | 0 (0.0%) | 0 (0.0%) | 0 (0.0%) |
| ***MYC*** | 3 (4.1%) | 0 (0.0%) | 3 (4.1%) | 0 (0.0%) | 0 (0.0%) | 0 (0.0%) | 0 (0.0%) |
| ***NOTCH1*** | 3 (4.1%) | 3 (4.1%) | 0 (0.0%) | 0 (0.0%) | 0 (0.0%) | 0 (0.0%) | 0 (0.0%) |
| ***SMAD2*** | 3 (4.1%) | 1 (1.4%) | 0 (0.0%) | 2 (2.7%) | 0 (0.0%) | 0 (0.0%) | 0 (0.0%) |
| ***SOX2*** | 3 (4.1%) | 0 (0.0%) | 3 (4.1%) | 0 (0.0%) | 0 (0.0%) | 0 (0.0%) | 0 (0.0%) |
| ***STK11*** | 3 (4.1%) | 3 (4.1%) | 0 (0.0%) | 0 (0.0%) | 0 (0.0%) | 0 (0.0%) | 0 (0.0%) |
| ***TERT*** | 3 (4.1%) | 3 (4.1%) | 0 (0.0%) | 0 (0.0%) | 0 (0.0%) | 0 (0.0%) | 0 (0.0%) |
| ***ASXL1*** | 2 (2.7%) | 2 (2.7%) | 0 (0.0%) | 0 (0.0%) | 0 (0.0%) | 0 (0.0%) | 0 (0.0%) |
| ***BCORL1*** | 2 (2.7%) | 2 (2.7%) | 0 (0.0%) | 0 (0.0%) | 0 (0.0%) | 0 (0.0%) | 0 (0.0%) |
| ***CBL*** | 2 (2.7%) | 2 (2.7%) | 0 (0.0%) | 0 (0.0%) | 0 (0.0%) | 0 (0.0%) | 0 (0.0%) |
| ***CCND2*** | 2 (2.7%) | 0 (0.0%) | 2 (2.7%) | 0 (0.0%) | 0 (0.0%) | 0 (0.0%) | 0 (0.0%) |
| ***CD274 (PD-L1)*** | 2 (2.7%) | 0 (0.0%) | 2 (2.7%) | 0 (0.0%) | 0 (0.0%) | 0 (0.0%) | 0 (0.0%) |
| ***CDH1*** | 2 (2.7%) | 2 (2.7%) | 0 (0.0%) | 0 (0.0%) | 0 (0.0%) | 0 (0.0%) | 0 (0.0%) |
| ***CDK4*** | 2 (2.7%) | 1 (1.4%) | 1 (1.4%) | 0 (0.0%) | 0 (0.0%) | 0 (0.0%) | 0 (0.0%) |
| ***CDKN1B*** | 2 (2.7%) | 2 (2.7%) | 0 (0.0%) | 0 (0.0%) | 0 (0.0%) | 0 (0.0%) | 0 (0.0%) |
| ***CTNNA1*** | 2 (2.7%) | 1 (1.4%) | 0 (0.0%) | 0 (0.0%) | 0 (0.0%) | 1 (1.4%) | 0 (0.0%) |
| ***DNMT3A*** | 2 (2.7%) | 2 (2.7%) | 0 (0.0%) | 0 (0.0%) | 0 (0.0%) | 0 (0.0%) | 0 (0.0%) |
| ***FAT1*** | 2 (2.7%) | 2 (2.7%) | 0 (0.0%) | 0 (0.0%) | 0 (0.0%) | 0 (0.0%) | 0 (0.0%) |
| ***FBXW7*** | 2 (2.7%) | 2 (2.7%) | 0 (0.0%) | 0 (0.0%) | 0 (0.0%) | 0 (0.0%) | 0 (0.0%) |
| ***FGF19*** | 2 (2.7%) | 0 (0.0%) | 2 (2.7%) | 0 (0.0%) | 0 (0.0%) | 0 (0.0%) | 0 (0.0%) |
| ***FGF3*** | 2 (2.7%) | 0 (0.0%) | 2 (2.7%) | 0 (0.0%) | 0 (0.0%) | 0 (0.0%) | 0 (0.0%) |
| ***FGF4*** | 2 (2.7%) | 0 (0.0%) | 2 (2.7%) | 0 (0.0%) | 0 (0.0%) | 0 (0.0%) | 0 (0.0%) |
| ***FGFR1*** | 2 (2.7%) | 0 (0.0%) | 2 (2.7%) | 0 (0.0%) | 0 (0.0%) | 0 (0.0%) | 0 (0.0%) |
| ***INHBA*** | 2 (2.7%) | 2 (2.7%) | 0 (0.0%) | 0 (0.0%) | 0 (0.0%) | 0 (0.0%) | 0 (0.0%) |
| ***JAK2*** | 2 (2.7%) | 0 (0.0%) | 2 (2.7%) | 0 (0.0%) | 0 (0.0%) | 0 (0.0%) | 0 (0.0%) |
| ***KDM5A*** | 2 (2.7%) | 0 (0.0%) | 2 (2.7%) | 0 (0.0%) | 0 (0.0%) | 0 (0.0%) | 0 (0.0%) |
| ***KDM6A*** | 2 (2.7%) | 0 (0.0%) | 0 (0.0%) | 1 (1.4%) | 0 (0.0%) | 1 (1.4%) | 0 (0.0%) |
| ***MLH1*** | 2 (2.7%) | 1 (1.4%) | 0 (0.0%) | 0 (0.0%) | 0 (0.0%) | 1 (1.4%) | 0 (0.0%) |
| ***MYCL1*** | 2 (2.7%) | 0 (0.0%) | 2 (2.7%) | 0 (0.0%) | 0 (0.0%) | 0 (0.0%) | 0 (0.0%) |
| ***NRAS*** | 2 (2.7%) | 2 (2.7%) | 0 (0.0%) | 0 (0.0%) | 0 (0.0%) | 0 (0.0%) | 0 (0.0%) |
| ***PBRM1*** | 2 (2.7%) | 2 (2.7%) | 0 (0.0%) | 0 (0.0%) | 0 (0.0%) | 0 (0.0%) | 0 (0.0%) |
| ***RBM10*** | 2 (2.7%) | 2 (2.7%) | 0 (0.0%) | 0 (0.0%) | 0 (0.0%) | 0 (0.0%) | 0 (0.0%) |
| ***RNF43*** | 2 (2.7%) | 2 (2.7%) | 0 (0.0%) | 0 (0.0%) | 0 (0.0%) | 0 (0.0%) | 0 (0.0%) |
| ***SOX9*** | 2 (2.7%) | 2 (2.7%) | 0 (0.0%) | 0 (0.0%) | 0 (0.0%) | 0 (0.0%) | 0 (0.0%) |
| ***SPTA1*** | 2 (2.7%) | 2 (2.7%) | 0 (0.0%) | 0 (0.0%) | 0 (0.0%) | 0 (0.0%) | 0 (0.0%) |
| ***TERC*** | 2 (2.7%) | 0 (0.0%) | 2 (2.7%) | 0 (0.0%) | 0 (0.0%) | 0 (0.0%) | 0 (0.0%) |
| ***TET2*** | 2 (2.7%) | 2 (2.7%) | 0 (0.0%) | 0 (0.0%) | 0 (0.0%) | 0 (0.0%) | 0 (0.0%) |
| ***AKT1*** | 1 (1.4%) | 1 (1.4%) | 0 (0.0%) | 0 (0.0%) | 0 (0.0%) | 0 (0.0%) | 0 (0.0%) |
| ***AKT2*** | 1 (1.4%) | 0 (0.0%) | 1 (1.4%) | 0 (0.0%) | 0 (0.0%) | 0 (0.0%) | 0 (0.0%) |
| ***ALK*** | 1 (1.4%) | 0 (0.0%) | 0 (0.0%) | 0 (0.0%) | 1 (1.4%) | 0 (0.0%) | 0 (0.0%) |
| ***ARAF*** | 1 (1.4%) | 1 (1.4%) | 0 (0.0%) | 0 (0.0%) | 0 (0.0%) | 0 (0.0%) | 0 (0.0%) |
| ***ARID2*** | 1 (1.4%) | 1 (1.4%) | 0 (0.0%) | 0 (0.0%) | 0 (0.0%) | 0 (0.0%) | 0 (0.0%) |
| ***ATRX*** | 1 (1.4%) | 0 (0.0%) | 0 (0.0%) | 0 (0.0%) | 0 (0.0%) | 1 (1.4%) | 0 (0.0%) |
| ***AXL*** | 1 (1.4%) | 0 (0.0%) | 1 (1.4%) | 0 (0.0%) | 0 (0.0%) | 0 (0.0%) | 0 (0.0%) |
| ***BARD1*** | 1 (1.4%) | 1 (1.4%) | 0 (0.0%) | 0 (0.0%) | 0 (0.0%) | 0 (0.0%) | 0 (0.0%) |
| ***BRCA1*** | 1 (1.4%) | 0 (0.0%) | 0 (0.0%) | 0 (0.0%) | 0 (0.0%) | 1 (1.4%) | 0 (0.0%) |
| ***BRCA2*** | 1 (1.4%) | 1 (1.4%) | 0 (0.0%) | 0 (0.0%) | 0 (0.0%) | 0 (0.0%) | 0 (0.0%) |
| ***BRD4*** | 1 (1.4%) | 0 (0.0%) | 0 (0.0%) | 0 (0.0%) | 0 (0.0%) | 0 (0.0%) | 1 (1.4%) |
| ***BRIP1*** | 1 (1.4%) | 1 (1.4%) | 0 (0.0%) | 0 (0.0%) | 0 (0.0%) | 0 (0.0%) | 0 (0.0%) |
| ***CDK12*** | 1 (1.4%) | 1 (1.4%) | 0 (0.0%) | 0 (0.0%) | 0 (0.0%) | 0 (0.0%) | 0 (0.0%) |
| ***CHD2*** | 1 (1.4%) | 1 (1.4%) | 0 (0.0%) | 0 (0.0%) | 0 (0.0%) | 0 (0.0%) | 0 (0.0%) |
| ***CIC*** | 1 (1.4%) | 1 (1.4%) | 0 (0.0%) | 0 (0.0%) | 0 (0.0%) | 0 (0.0%) | 0 (0.0%) |
| ***CREBBP*** | 1 (1.4%) | 1 (1.4%) | 0 (0.0%) | 0 (0.0%) | 0 (0.0%) | 0 (0.0%) | 0 (0.0%) |
| ***CSF1R*** | 1 (1.4%) | 1 (1.4%) | 0 (0.0%) | 0 (0.0%) | 0 (0.0%) | 0 (0.0%) | 0 (0.0%) |
| ***CTCF*** | 1 (1.4%) | 1 (1.4%) | 0 (0.0%) | 0 (0.0%) | 0 (0.0%) | 0 (0.0%) | 0 (0.0%) |
| ***ETV6*** | 1 (1.4%) | 0 (0.0%) | 0 (0.0%) | 0 (0.0%) | 0 (0.0%) | 1 (1.4%) | 0 (0.0%) |
| ***FANCA*** | 1 (1.4%) | 1 (1.4%) | 0 (0.0%) | 0 (0.0%) | 0 (0.0%) | 0 (0.0%) | 0 (0.0%) |
| ***FANCC*** | 1 (1.4%) | 0 (0.0%) | 0 (0.0%) | 0 (0.0%) | 0 (0.0%) | 1 (1.4%) | 0 (0.0%) |
| ***FGF10*** | 1 (1.4%) | 0 (0.0%) | 1 (1.4%) | 0 (0.0%) | 0 (0.0%) | 0 (0.0%) | 0 (0.0%) |
| ***FGF23*** | 1 (1.4%) | 0 (0.0%) | 1 (1.4%) | 0 (0.0%) | 0 (0.0%) | 0 (0.0%) | 0 (0.0%) |
| ***FGF6*** | 1 (1.4%) | 0 (0.0%) | 1 (1.4%) | 0 (0.0%) | 0 (0.0%) | 0 (0.0%) | 0 (0.0%) |
| ***FGFR2*** | 1 (1.4%) | 0 (0.0%) | 0 (0.0%) | 0 (0.0%) | 1 (1.4%) | 0 (0.0%) | 0 (0.0%) |
| ***FLCN*** | 1 (1.4%) | 1 (1.4%) | 0 (0.0%) | 0 (0.0%) | 0 (0.0%) | 0 (0.0%) | 0 (0.0%) |
| ***FLT4*** | 1 (1.4%) | 0 (0.0%) | 1 (1.4%) | 0 (0.0%) | 0 (0.0%) | 0 (0.0%) | 0 (0.0%) |
| ***GABRA6*** | 1 (1.4%) | 1 (1.4%) | 0 (0.0%) | 0 (0.0%) | 0 (0.0%) | 0 (0.0%) | 0 (0.0%) |
| ***GNA11*** | 1 (1.4%) | 1 (1.4%) | 0 (0.0%) | 0 (0.0%) | 0 (0.0%) | 0 (0.0%) | 0 (0.0%) |
| ***HNF1A*** | 1 (1.4%) | 1 (1.4%) | 0 (0.0%) | 0 (0.0%) | 0 (0.0%) | 0 (0.0%) | 0 (0.0%) |
| ***HRAS*** | 1 (1.4%) | 1 (1.4%) | 0 (0.0%) | 0 (0.0%) | 0 (0.0%) | 0 (0.0%) | 0 (0.0%) |
| ***IDH1*** | 1 (1.4%) | 1 (1.4%) | 0 (0.0%) | 0 (0.0%) | 0 (0.0%) | 0 (0.0%) | 0 (0.0%) |
| ***JAK1*** | 1 (1.4%) | 1 (1.4%) | 0 (0.0%) | 0 (0.0%) | 0 (0.0%) | 0 (0.0%) | 0 (0.0%) |
| ***KEAP1*** | 1 (1.4%) | 1 (1.4%) | 0 (0.0%) | 0 (0.0%) | 0 (0.0%) | 0 (0.0%) | 0 (0.0%) |
| ***KIT*** | 1 (1.4%) | 0 (0.0%) | 0 (0.0%) | 0 (0.0%) | 0 (0.0%) | 1 (1.4%) | 0 (0.0%) |
| ***LRP18*** | 1 (1.4%) | 1 (1.4%) | 0 (0.0%) | 0 (0.0%) | 0 (0.0%) | 0 (0.0%) | 0 (0.0%) |
| ***LZTR1*** | 1 (1.4%) | 1 (1.4%) | 0 (0.0%) | 0 (0.0%) | 0 (0.0%) | 0 (0.0%) | 0 (0.0%) |
| ***MAGI2*** | 1 (1.4%) | 1 (1.4%) | 0 (0.0%) | 0 (0.0%) | 0 (0.0%) | 0 (0.0%) | 0 (0.0%) |
| ***MAP2K1*** | 1 (1.4%) | 1 (1.4%) | 0 (0.0%) | 0 (0.0%) | 0 (0.0%) | 0 (0.0%) | 0 (0.0%) |
| ***MAP2K4*** | 1 (1.4%) | 1 (1.4%) | 0 (0.0%) | 0 (0.0%) | 0 (0.0%) | 0 (0.0%) | 0 (0.0%) |
| ***MEN1*** | 1 (1.4%) | 1 (1.4%) | 0 (0.0%) | 0 (0.0%) | 0 (0.0%) | 0 (0.0%) | 0 (0.0%) |
| ***MLL3*** | 1 (1.4%) | 1 (1.4%) | 0 (0.0%) | 0 (0.0%) | 0 (0.0%) | 0 (0.0%) | 0 (0.0%) |
| ***MSH6*** | 1 (1.4%) | 1 (1.4%) | 0 (0.0%) | 0 (0.0%) | 0 (0.0%) | 0 (0.0%) | 0 (0.0%) |
| ***MYST3*** | 1 (1.4%) | 0 (0.0%) | 1 (1.4%) | 0 (0.0%) | 0 (0.0%) | 0 (0.0%) | 0 (0.0%) |
| ***NOTCH2*** | 1 (1.4%) | 1 (1.4%) | 0 (0.0%) | 0 (0.0%) | 0 (0.0%) | 0 (0.0%) | 0 (0.0%) |
| ***NSD1*** | 1 (1.4%) | 1 (1.4%) | 0 (0.0%) | 0 (0.0%) | 0 (0.0%) | 0 (0.0%) | 0 (0.0%) |
| ***PALB2*** | 1 (1.4%) | 1 (1.4%) | 0 (0.0%) | 0 (0.0%) | 0 (0.0%) | 0 (0.0%) | 0 (0.0%) |
| ***PARK2*** | 1 (1.4%) | 0 (0.0%) | 0 (0.0%) | 1 (1.4%) | 0 (0.0%) | 0 (0.0%) | 0 (0.0%) |
| ***PDCD1LG2 (PD-L2)*** | 1 (1.4%) | 0 (0.0%) | 1 (1.4%) | 0 (0.0%) | 0 (0.0%) | 0 (0.0%) | 0 (0.0%) |
| ***PDGFRA*** | 1 (1.4%) | 1 (1.4%) | 0 (0.0%) | 0 (0.0%) | 0 (0.0%) | 0 (0.0%) | 0 (0.0%) |
| ***PIK3CB*** | 1 (1.4%) | 1 (1.4%) | 0 (0.0%) | 0 (0.0%) | 0 (0.0%) | 0 (0.0%) | 0 (0.0%) |
| ***PRKCI*** | 1 (1.4%) | 0 (0.0%) | 1 (1.4%) | 0 (0.0%) | 0 (0.0%) | 0 (0.0%) | 0 (0.0%) |
| ***PTCH1*** | 1 (1.4%) | 1 (1.4%) | 0 (0.0%) | 0 (0.0%) | 0 (0.0%) | 0 (0.0%) | 0 (0.0%) |
| ***RANBP2*** | 1 (1.4%) | 1 (1.4%) | 0 (0.0%) | 0 (0.0%) | 0 (0.0%) | 0 (0.0%) | 0 (0.0%) |
| ***RET*** | 1 (1.4%) | 0 (0.0%) | 0 (0.0%) | 0 (0.0%) | 1 (1.4%) | 0 (0.0%) | 0 (0.0%) |
| ***RICTOR*** | 1 (1.4%) | 0 (0.0%) | 1 (1.4%) | 0 (0.0%) | 0 (0.0%) | 0 (0.0%) | 0 (0.0%) |
| ***RPTOR*** | 1 (1.4%) | 0 (0.0%) | 1 (1.4%) | 0 (0.0%) | 0 (0.0%) | 0 (0.0%) | 0 (0.0%) |
| ***RUNX1*** | 1 (1.4%) | 1 (1.4%) | 0 (0.0%) | 0 (0.0%) | 0 (0.0%) | 0 (0.0%) | 0 (0.0%) |
| ***SDHB*** | 1 (1.4%) | 1 (1.4%) | 0 (0.0%) | 0 (0.0%) | 0 (0.0%) | 0 (0.0%) | 0 (0.0%) |
| ***SPEN*** | 1 (1.4%) | 1 (1.4%) | 0 (0.0%) | 0 (0.0%) | 0 (0.0%) | 0 (0.0%) | 0 (0.0%) |
| ***STAG2*** | 1 (1.4%) | 0 (0.0%) | 0 (0.0%) | 1 (1.4%) | 0 (0.0%) | 0 (0.0%) | 0 (0.0%) |
| ***TSC2*** | 1 (1.4%) | 0 (0.0%) | 0 (0.0%) | 0 (0.0%) | 0 (0.0%) | 1 (1.4%) | 0 (0.0%) |

Numbers presented are numbers of patients and percent of total (N=74) patients with the specified alteration.

**Supplemental Table 2**. Blood-derived cfDNA analysis in patients with carcinoma of unknown primary (N = 72).

|  | **Characterized alterations**  **No. (%)** | **Mutations**  **No. (%)** | **Amplification**  **No. (%)** | **Loss No. (%)** | **Fusion/ rearrangement**  **No. (%)** | **Indel**  **No. (%)** | **Multiple alterations**  **No. (%)** |
| --- | --- | --- | --- | --- | --- | --- | --- |
| ***TP53*** | 44 (61.1%) | 43 (59.7%) | 0 (0.0%) | 0 (0.0%) | 0 (0.0%) | 0 (0.0%) | 1 (1.4%) |
| ***KRAS*** | 15 (20.8%) | 12 (16.7%) | 2 (2.8%) | 0 (0.0%) | 0 (0.0%) | 0 (0.0%) | 1 (1.4%) |
| ***PIK3CA*** | 12 (16.7%) | 8 (11.1%) | 4 (5.6%) | 0 (0.0%) | 0 (0.0%) | 0 (0.0%) | 0 (0.0%) |
| ***NF1*** | 9 (12.5%) | 7 (9.7%) | 0 (0.0%) | 0 (0.0%) | 0 (0.0%) | 2 (2.8%) | 0 (0.0%) |
| ***ERBB2*** | 8 (11.1%) | 2 (2.8%) | 4 (5.6%) | 0 (0.0%) | 0 (0.0%) | 0 (0.0%) | 2 (2.8%) |
| ***EGFR*** | 7 (9.7%) | 2 (2.8%) | 5 (6.9%) | 0 (0.0%) | 0 (0.0%) | 0 (0.0%) | 0 (0.0%) |
| ***MYC*** | 6 (8.3%) | 0 (0.0%) | 6 (8.3%) | 0 (0.0%) | 0 (0.0%) | 0 (0.0%) | 0 (0.0%) |
| ***BRAF*** | 5 (6.9%) | 1 (1.4%) | 3 (4.2%) | 0 (0.0%) | 0 (0.0%) | 0 (0.0%) | 1 (1.4%) |
| ***MET*** | 5 (6.9%) | 1 (1.4%) | 4 (5.6%) | 0 (0.0%) | 0 (0.0%) | 0 (0.0%) | 0 (0.0%) |
| ***APC*** | 3 (4.2%) | 3 (4.2%) | 0 (0.0%) | 0 (0.0%) | 0 (0.0%) | 0 (0.0%) | 0 (0.0%) |
| ***ARID1A*** | 3 (4.2%) | 3 (4.2%) | 0 (0.0%) | 0 (0.0%) | 0 (0.0%) | 0 (0.0%) | 0 (0.0%) |
| ***BRCA1*** | 3 (4.2%) | 3 (4.2%) | 0 (0.0%) | 0 (0.0%) | 0 (0.0%) | 0 (0.0%) | 0 (0.0%) |
| ***FGFR1*** | 3 (4.2%) | 0 (0.0%) | 3 (4.2%) | 0 (0.0%) | 0 (0.0%) | 0 (0.0%) | 0 (0.0%) |
| ***FGFR3*** | 3 (4.2%) | 2 (2.8%) | 0 (0.0%) | 0 (0.0%) | 1 (1.4%) | 0 (0.0%) | 0 (0.0%) |
| ***GNAS*** | 3 (4.2%) | 3 (4.2%) | 0 (0.0%) | 0 (0.0%) | 0 (0.0%) | 0 (0.0%) | 0 (0.0%) |
| ***PTEN*** | 3 (4.2%) | 3 (4.2%) | 0 (0.0%) | 0 (0.0%) | 0 (0.0%) | 0 (0.0%) | 0 (0.0%) |
| ***ATM*** | 2 (2.8%) | 2 (2.8%) | 0 (0.0%) | 0 (0.0%) | 0 (0.0%) | 0 (0.0%) | 0 (0.0%) |
| ***CCND2*** | 2 (2.8%) | 1 (1.4%) | 1 (1.4%) | 0 (0.0%) | 0 (0.0%) | 0 (0.0%) | 0 (0.0%) |
| ***FBXW7*** | 2 (2.8%) | 2 (2.8%) | 0 (0.0%) | 0 (0.0%) | 0 (0.0%) | 0 (0.0%) | 0 (0.0%) |
| ***NRAS*** | 2 (2.8%) | 2 (2.8%) | 0 (0.0%) | 0 (0.0%) | 0 (0.0%) | 0 (0.0%) | 0 (0.0%) |
| ***RAF1*** | 2 (2.8%) | 1 (1.4%) | 1 (1.4%) | 0 (0.0%) | 0 (0.0%) | 0 (0.0%) | 0 (0.0%) |
| ***RB1*** | 2 (2.8%) | 2 (2.8%) | 0 (0.0%) | 0 (0.0%) | 0 (0.0%) | 0 (0.0%) | 0 (0.0%) |
| ***STK11*** | 2 (2.8%) | 2 (2.8%) | 0 (0.0%) | 0 (0.0%) | 0 (0.0%) | 0 (0.0%) | 0 (0.0%) |
| ***AKT1*** | 1 (1.4%) | 1 (1.4%) | 0 (0.0%) | 0 (0.0%) | 0 (0.0%) | 0 (0.0%) | 0 (0.0%) |
| ***ARAF*** | 1 (1.4%) | 1 (1.4%) | 0 (0.0%) | 0 (0.0%) | 0 (0.0%) | 0 (0.0%) | 0 (0.0%) |
| ***BRCA2*** | 1 (1.4%) | 1 (1.4%) | 0 (0.0%) | 0 (0.0%) | 0 (0.0%) | 0 (0.0%) | 0 (0.0%) |
| ***CCNE1*** | 1 (1.4%) | 0 (0.0%) | 1 (1.4%) | 0 (0.0%) | 0 (0.0%) | 0 (0.0%) | 0 (0.0%) |
| ***CDK4*** | 1 (1.4%) | 1 (1.4%) | 0 (0.0%) | 0 (0.0%) | 0 (0.0%) | 0 (0.0%) | 0 (0.0%) |
| ***CDK6*** | 1 (1.4%) | 0 (0.0%) | 1 (1.4%) | 0 (0.0%) | 0 (0.0%) | 0 (0.0%) | 0 (0.0%) |
| ***CDKN2A*** | 1 (1.4%) | 1 (1.4%) | 0 (0.0%) | 0 (0.0%) | 0 (0.0%) | 0 (0.0%) | 0 (0.0%) |
| ***CTNNB1*** | 1 (1.4%) | 1 (1.4%) | 0 (0.0%) | 0 (0.0%) | 0 (0.0%) | 0 (0.0%) | 0 (0.0%) |
| ***ERRFI1*** | 1 (1.4%) | 1 (1.4%) | 0 (0.0%) | 0 (0.0%) | 0 (0.0%) | 0 (0.0%) | 0 (0.0%) |
| ***ESR1*** | 1 (1.4%) | 1 (1.4%) | 0 (0.0%) | 0 (0.0%) | 0 (0.0%) | 0 (0.0%) | 0 (0.0%) |
| ***GNA11*** | 1 (1.4%) | 1 (1.4%) | 0 (0.0%) | 0 (0.0%) | 0 (0.0%) | 0 (0.0%) | 0 (0.0%) |
| ***MLH1*** | 1 (1.4%) | 1 (1.4%) | 0 (0.0%) | 0 (0.0%) | 0 (0.0%) | 0 (0.0%) | 0 (0.0%) |
| ***MTOR*** | 1 (1.4%) | 1 (1.4%) | 0 (0.0%) | 0 (0.0%) | 0 (0.0%) | 0 (0.0%) | 0 (0.0%) |
| ***NFE2L2*** | 1 (1.4%) | 1 (1.4%) | 0 (0.0%) | 0 (0.0%) | 0 (0.0%) | 0 (0.0%) | 0 (0.0%) |
| ***PDGFRA*** | 1 (1.4%) | 1 (1.4%) | 0 (0.0%) | 0 (0.0%) | 0 (0.0%) | 0 (0.0%) | 0 (0.0%) |
| ***RET*** | 1 (1.4%) | 0 (0.0%) | 0 (0.0%) | 0 (0.0%) | 1 (1.4%) | 0 (0.0%) | 0 (0.0%) |
| ***SMAD4*** | 1 (1.4%) | 1 (1.4%) | 0 (0.0%) | 0 (0.0%) | 0 (0.0%) | 0 (0.0%) | 0 (0.0%) |

Numbers presented are numbers of patients and percent of total (N=72) patients with the specified alteration.

**Supplemental Table 3**. Immune-profiling with RNA sequencing in patients with carcinoma of unknown primary (N = 12).

| **Checkpoint markers** | Low/very low (Number) | Moderate  (Number) | High/ very high  (Number) |
| --- | --- | --- | --- |
| PD-1 | 8 | 3 | 1 |
| PD-L1 | 7 | 3 | 2 |
| PD-L2 | 4 | 7 | 1 |
| **Other checkpoint markers** |  |  |  |
| BTLA | 11 | 1 | 0 |
| CTLA-4 | 9 | 3 | 0 |
| LAG3 | 8 | 3 | 1 |
| TIM3 | 5 | 7 | 0 |
| VISTA | 5 | 7 | 0 |
| TNFRSF14 | 9 | 3 | 0 |
| **Myeloid suppression markers** |  |  |  |
| CCL2 | 5 | 6 | 1 |
| CCR2 | 8 | 3 | 1 |
| CD163 | 7 | 5 | 0 |
| CD68 | 6 | 3 | 3 |
| CSF1R | 4 | 6 | 2 |
| **Metabolic immune escape markers** |  |  |  |
| ADORA2A | 7 | 4 | 1 |
| CD39 | 6 | 5 | 1 |
| IDO1 | 9 | 2 | 1 |
| **Anti-inflammatory response** **markers** |  |  |  |
| IL10 | 6 | 6 | 0 |
| TGFB1 | 2 | 8 | 2 |
| **T-cell primed makers** |  |  |  |
| CD137 | 8 | 4 | 0 |
| CD27 | 8 | 3 | 1 |
| CD28 | 8 | 3 | 1 |
| CD40 | 6 | 6 | 0 |
| CD40 ligand | 9 | 2 | 1 |
| GITR | 6 | 5 | 1 |
| ICOS | 7 | 4 | 1 |
| ICOS ligand | 6 | 3 | 3 |
| OX40 | 8 | 3 | 1 |
| OX40 ligand | 8 | 3 | 1 |
| GZMB | 8 | 3 | 1 |
| IFNG | 8 | 3 | 1 |
| CD80 (B7-1) | 8 | 4 | 0 |
| CD86 (B7-2) | 6 | 6 | 0 |
| TBX21 | 9 | 3 | 0 |
| **Pro-inflammatory response markers** |  |  |  |
| IL1B | 6 | 4 | 2 |
| STAT1 | 5 | 6 | 1 |
| TNF | 5 | 6 | 1 |
| DDX58 | 2 | 7 | 3 |
| MX1 | 5 | 6 | 1 |
| CXCL10 | 7 | 2 | 3 |
| CXCR6 | 8 | 2 | 2 |
| **Tumor infiltrating lymphocytes markers** |  |  |  |
| CD2 | 4 | 7 | 1 |
| CD3 | 4 | 5 | 3 |
| CD4 | 2 | 5 | 5 |
| CD8 | 6 | 5 | 1 |
| FOXP3 | 6 | 2 | 4 |
| KLRD1 | 4 | 7 | 1 |
| SLAMF4 | 6 | 4 | 2 |
| CD20 | 7 | 4 | 1 |
| **Other markers** |  |  |  |
| CD38 | 7 | 5 | 0 |
| GATA3 | 8 | 2 | 2 |

Numbers shown are number of patients with the alteration.

**Supplemental Table** **4.** OncoKB annotation and level of evidence ([www.oncokb.org](http://www.oncokb.org/)). (accessed as of July 17, 2020)

| **OncoKB annotation** | **Comment** | **List of alterations** |
| --- | --- | --- |
| Level 1 alteration | FDA-recognized biomarker predictive of response to an FDA-approved drug in this indication. | Supplemental Table 5 |
| Level 2 alteration | Standard care biomarker recommended by the NCCN or other expert panels predictive of response to an FDA-approved drug in this indication. | Supplemental Table 5 |
| Resistance R1 alteration | Standard care biomarker predictive of resistance to an FDA-approved drug in this indication | Supplemental Table 5 |

**Abbreviation**: FDA, Food and Drug Administration; NCCN, National Comprehensive Cancer Network.

**Supplemental Table 5.** Alterations with level of evidence according to OncoKB annotation.

| **Gene** | **Alteration(s)** | **Cancer Type** | **Level of Evidence** |
| --- | --- | --- | --- |
| **Level 1** | | | |
| ***ALK*** | Fusions | NSCLC | 1 |
| ***ALK*** | Oncogenic alterations | NSCLC | 1 |
| **ATM** | Oncogenic alterations | Prostate | 1 |
| ***BRAF*** | V600E | Anaplastic thyroid cancer, CRC, melanoma | 1 |
| ***BRAF*** | V600K | Melanoma | 1 |
| ***BRCA1/2*** | Oncogenic alterations | Ovarian, peritoneal serous, prostate | 1 |
| ***CDK12*** | Oncogenic alterations | Prostate | 1 |
| ***EGFR*** | G719, T790M, exon 19 deletions, L858R, S7681I, L861Q, | NSCLC | 1 |
| ***ERBB2*** | Amplification | Breast, esophagastric | 1 |
| ***FGFR2/3*** | Fusions | Bladder, cholangiocarcinoma | 1 |
| ***FGFR3*** | G370C, R248C, S249C, Y373C | Bladder | 1 |
| ***KIT*** | Oncogenic alterations | GIST | 1 |
| ***NF1*** | Oncogenic alterations | Neurofibroma | 1 |
| ***MET*** | Exon 14 skipping alterations | NSCLC | 1 |
| ***NF1*** | Oncogenic alterations | Neurofibroma | 1 |
| ***NTRK1*** | Fusions | All solid tumors | 1 |
| **Not applicable** | Microsatellite instability – high | All solid tumors | 1 |
| ***PDGFRA*** | D842V, D842Y, D842_H845del, D842_H845insV | GIST | 1 |
| ***PIK3CA*** | Oncogenic alterations | Breast | 1 |
| ***RET*** | Fusions | NSCLC, thyroid | 1 |
| ***ROS1*** | Fusions | NSCLC | 1 |
| ***TSC1*** | Oncogenic alterations | CNS cancer | 1 |
| **Level 2** | | | |
| ***BRAF*** | V600E | CRC, astrocytoma, ganglioglioma, pleomorphic xanthoastrocytoma | 2 |
| ***BRAF*** | V600 | Melanoma, anaplastic thyroid | 2 |
| ***BRCA1/2*** | Oncogenic alterations | Breast, ovarian, peritoneal serous | 2 |
| ***CDK4*** | Amplification | Liposarcoma | 2 |
| ***EGFR*** | A763_Y764insFQEA | NSCLC | 2 |
| ***ERBB2*** | Oncogenic alterations | NSCLC | 2 |
| ***ERBB2*** | Amplification | CRC, uterine | 2 |
| ***KIT*** | Oncogenic alterations | GIST, melanoma, thymic | 2 |
| ***MET*** | D1010, exon 14 deletion, exon 14 splice mutation, Y1003 | NSCLC | 2 |
| ***MET*** | Amplification | NSCLC, RCC | 2 |
| ***PDGFRA*** | Oncogenic alterations | GIST | 2 |
| **Resistance R1** | | | |
| ***EGFR*** | Exon 20 insertions | NSCLC | R1 |
| ***EGFR*** | T790M | NSCLC | R1 |
| ***KRAS*** | Oncogenic alterations | CRC | R1 |
| ***NRAS*** | Oncogenic alterations | CRC | R1 |
| ***PDGFRA*** | D842V | GIST | R1 |

**Supplemental Table 6**. Details of the 7 patients with high matching score (>50) who achieved partial response.


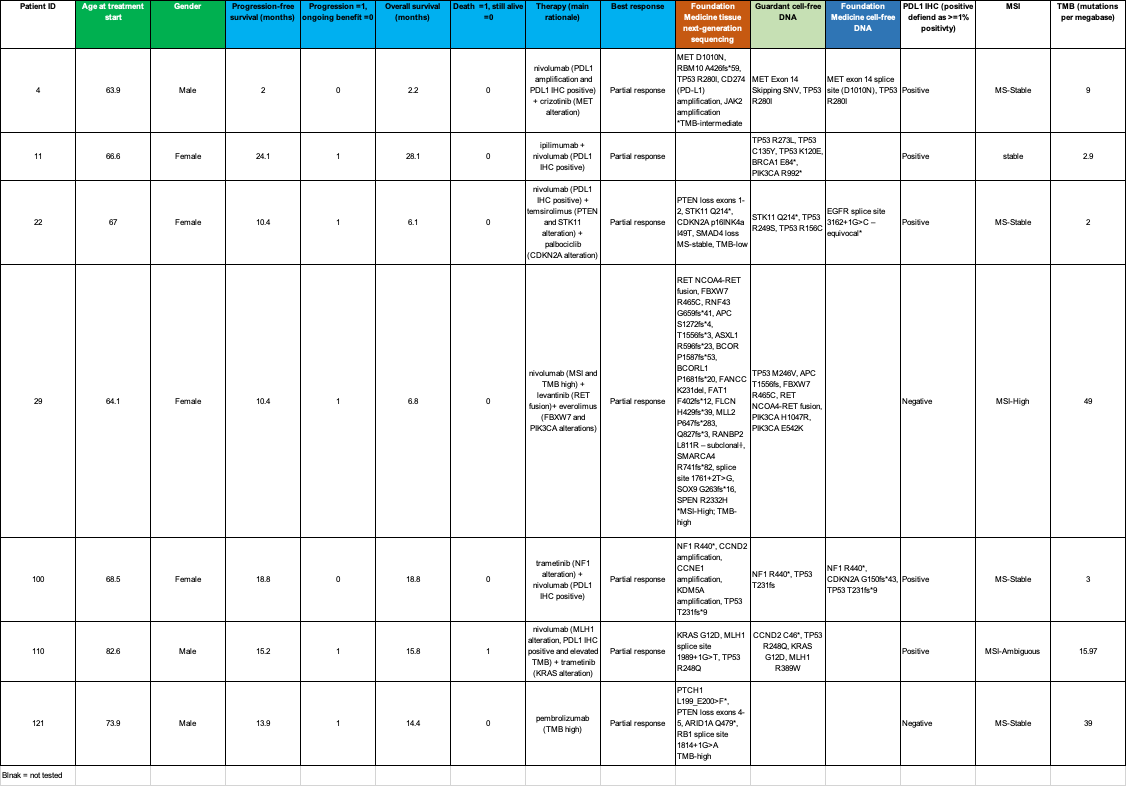


**Supplemental Figure 1**. CONSORT diagram of the study.

6,497 patients from the PREDICT database

97 patients with CUP

62 evaluable

treated CUP patients

47 patients with low degrees of matching to therapy (Matching Score≤50%)

15 patients with high degrees of matching to therapy (Matching Score>50%)

**Supplemental Figure 2.** The Venn diagram shows overlap of test types with further detail in the chart.


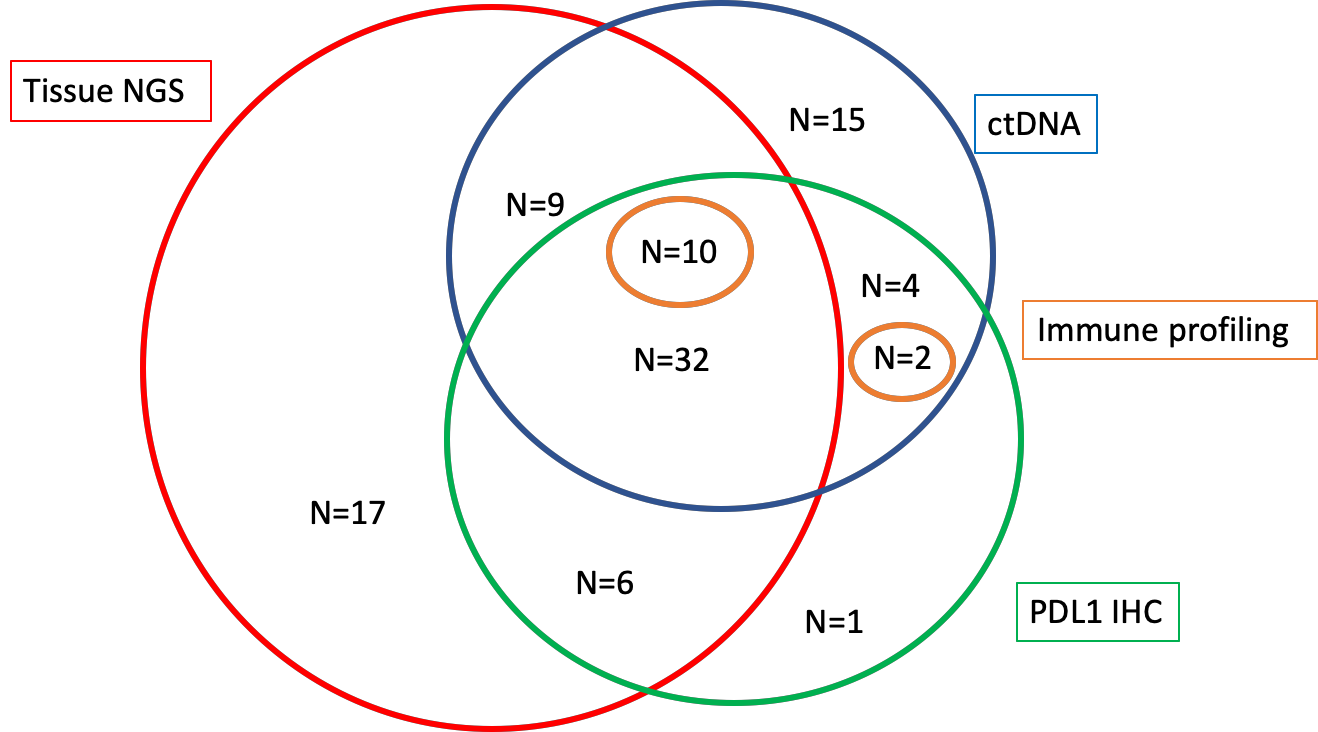


| **Profiling** | **Number of patients**  **being tested** |
| --- | --- |
| **Tissue NGS alone** | 17 |
| **ctDNA alone** | 15 |
| **PDL1 IHC alone** | 1 |
| **Immune profiling alone** | 0 |
| **Tissue NGS and ctDNA** | 9 |
| **Tissue NGS and PDL1 IHC** | 6 |
| **Tissue NGS and Immune profiling** | 0 |
| **ctDNA and PDL1 IHC** | 4 |
| **ctDNA and immune profioing** | 0 |
| **PDL1 IHC and Immune profilig** | 0 |
| **Tissue NGS, ctDNA, PDL1 IHC** | 32 |
| **ctDNA, PDL1 IHC, Immunue profiling** | 2 |
| **Tissue NGS, PDL1 IHC, Immune profiling** | 0 |
| **All 4 test types (Tissue NGS, ctDNA, PDL1 IHC, Immune profiling)** | 10 |
| **No testing** | 1 |
